# Innate immunity in allergy

**DOI:** 10.1111/all.13788

**Published:** 2019-04-14

**Authors:** Kazuhiko Maeda, Matias J. Caldez, Shizuo Akira

**Affiliations:** ^1^ Laboratory of Host Defense, The World Premier International Research Center Initiative (WPI) Immunology Frontier Research Center (IFReC) Osaka University Osaka Japan

**Keywords:** inflammasome, pattern recognition receptors, pyroptosis

## Abstract

Innate immune system quickly responds to invasion of microbes and foreign substances through the extracellular and intracellular sensing receptors, which recognize distinctive molecular and structural patterns. The recognition of innate immune receptors leads to the induction of inflammatory and adaptive immune responses by activating downstream signaling pathways. Allergy is an immune‐related disease and results from a hypersensitive immune response to harmless substances in the environment. However, less is known about the activation of innate immunity during exposure to allergens. New insights into the innate immune system by sensors and their signaling cascades provide us with more important clues and a framework for understanding allergy disorders. In this review, we will focus on recent advances in the innate immune sensing system.

## 1 INTRODUCTION

Our body is endlessly exposed to microbial agents and environmental noxious substances. These may cause serious illness, or toxicity to the body; therefore, they must be eliminated. This is mediated by the innate immune system, which is the first line of host defense against foreign invasion. Any disruption in the physical barriers that prevent pathogens from entering the body triggers pro‐inflammatory responses by activating myeloid cells and dendritic cells (DCs) that are central players of the innate immune defense. Furthermore, pro‐inflammatory responses induce antigen presentation shifting from an innate immune response to an acquired immune response. B cells and T cells, in which antigen receptors are individually specialized by DNA rearrangement, mainly mediate acquired immune responses. One of the main features making the innate immune system highly specialized is the germline‐encoded receptors distinguishing between self and nonself. This discrimination is mediated by membrane‐bound or cytoplasmic pattern recognition receptors (PRRs).^1^ The membrane‐bound receptors are Toll‐like receptors (TLRs) and C‐type lectin receptors (CLRs). The cytoplasmic‐type receptors are retinoic acid‐inducible gene I (RIG‐I)‐like receptors (RLRs) and nucleotide‐binding and oligomerization domain (NOD)‐like receptors (NLRs). These receptors directly sense various components from pathogens and distinguish conserved microbial structural features, called pathogen‐associated molecular patterns (PAMPs).^2^ The recognition of PAMPs leads to robust innate immune responses through the activation of these downstream signaling pathways. PRRs also recognize self‐components released from the damaged cells, called damage/danger‐associated molecular patterns (DAMPs), and can thus be associated with the pathogenesis of many diseases.

Allergy is an increasing problem in the health sector, with a soaring number of patients in recent years. Accumulating evidence suggests the importance of the innate immune system in the development of allergy susceptibility. Therefore, in this review, we will summarize recent advances in the involvement of PRRs in allergic diseases, and improvements to current allergy treatment modalities.

## 2 T‐HELPER 2 RESPONSE AND ALLERGY

In recent years, although highly criticized, the *hygiene hypothesis* has been proposed to explain the increase in frequency of patients with allergy worldwide.^3^ It suggests that a cleaner environment may lead to development of allergic diseases, highlighting that early exposure to microbes and parasites during childhood is essential to reduce development of susceptibility.^4^ As evidence in favor of the hygiene hypothesis, it is demonstrated that maternal intranasal exposure to the nonpathogenic microbe *Acinetobacter* protected against the development of experimental asthma in the progeny.^5^

Allergy is characterized by a T‐helper 2 (Th2) hypersensitivity response with a significant increase in immunoglobulin (Ig) E. Type 2 immune responses are characterized by the expression of type 2 cytokines, such as interleukin (IL)‐4, IL‐5, IL‐9, and IL‐13. Contact with bacteria during early development may be protective by inducing T‐helper 1 (Th1) cell differentiation. Intestinal microbiota also plays an important role in the prevention of allergies.^6^, ^7^ Although oral administration of ovalbumin (OVA) abrogates both Th1 and Th2 responses in specific pathogen‐free mice, only Th1 responses are reduced in germ‐free mice.^8^ The reconstitution of the intestinal microbiota of germ‐free mice suppressed the susceptibility of the Th2 responses in neonates,^9^ suggesting the importance of exposure to intestinal microbiota at the neonatal stage for prevention of allergies.

A high dose of lipopolysaccharide (LPS) promotes Th1 immune responses and prevents allergic disease in an OVA‐induced allergic asthma model.^10^ Conversely, a low amount of LPS skews the immune response to Th2 type and induces allergic airway inflammation in a thymic stromal lymphopoietin (TSLP)‐dependent manner.^11^ TSLP is an epithelial cell‐derived cytokine expressed in the thymus, lung, skin, and gut. The release of TSLP,^12^, ^13^ IL‐25,^14^, ^15^ IL‐33,^16^ and granulocyte‐macrophage colony‐stimulating factor (GM‐CSF)^17^ from nonlymphoid cells is important for the initiation of Th2 immune responses. Furthermore, epithelial cells in the damaged barriers may also cooperate in the initiation of Th2 immune responses to repair the tissue injury. Activation of group 2 innate lymphoid cells (ILC2s) by epithelium‐derived cytokines such as TSLP, IL‐7, IL‐25, IL‐33, and also IL‐4 induces the production of type 2 cytokines including IL‐5, IL‐9, IL‐13, and epidermal growth factor receptor (EGFR) ligand amphiregulin (AREG) for leading innate type 2 immunity.^18^, ^19^, ^20^ In this context, the intensity of activation of downstream signaling molecules and pathways may be strongly associated with the development of allergy.

## 3 TOLL‐LIKE RECEPTORS

The TLRs were the first discovered PRR and are mammalian homologues of *Drosophila* Toll protein involved in innate immune response.^21^, ^22^ Studies over the past two decades have revealed important roles of TLRs in a variety of biological phenomena including inflammation, the bridging between innate and acquired immune responses, and cancer cell proliferation and survival.

### 3.1 TLR family

**Figure 1:**
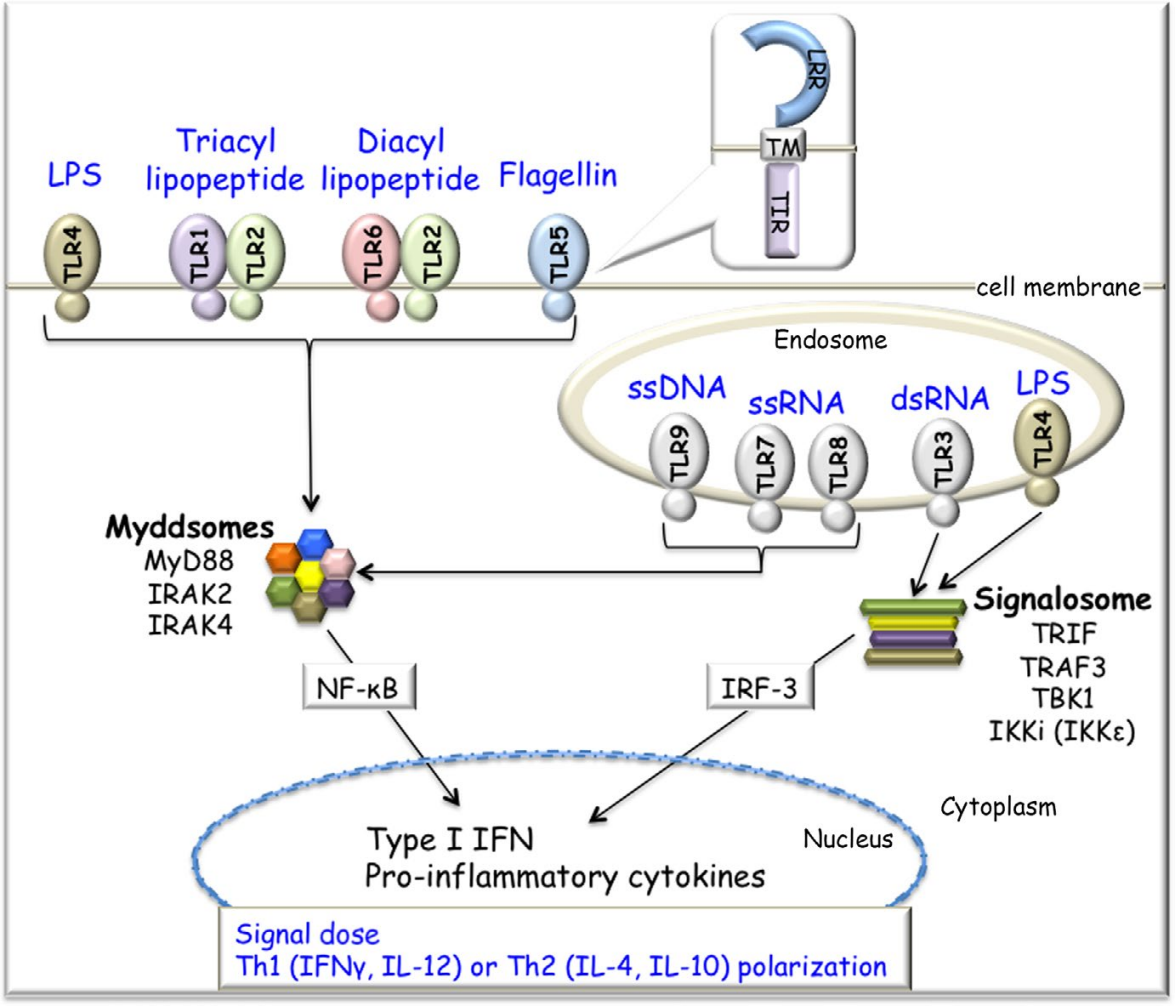
TLR‐mediated signaling pathway. All TLR proteins have LRR and TIR domains (in the balloon). Individual TLRs recognize different ligands, such as LPS, triacyl lipopeptide, diacyl lipopeptides, bacterial flagellin, DNA, and RNA. TLRs localize at the cell surface or in endosomes. TLRs recruit two adaptor proteins, MyD88 and TRIF. The ligand engagement of TLRs induces the formation of the Myddosome (MyD88 and IRAKs) and activates the NF‐κB pathway. TLR3 and TLR4 also induce the formation of a signalosome (TRIF, TRAF3, TBK1, and IKKi [IKKε]). Activated IRF‐3 induces type I IFN production. Th1/Th2 polarization into either a Th1 immune response or Th2 immune response is dependent on the signal dose through TLRs. IKKi, inducible inhibitor of NF‐κB (IκB) kinase; IRAK, IL‐1 receptor‐associated kinase; IRF‐3, IFN regulatory factor 3; LPS, lipopolysaccharide; LRR, leucine‐rich repeat; MyD88, myeloid differentiation primary response 88; NF‐κB, nuclear factor‐kappa B; TBK1, TRAF‐associated NF‐κB activator (TANK)‐binding kinase 1; Th1, T‐helper 1; Th2, T‐helper 2; TIR, Toll/interleukin‐1 receptor; TLR, Toll‐like receptor; TM, transmembrane; TRAF3, TNF receptor‐associated factor 3; TRIF, TIR‐domain‐containing adaptor inducing interferon (IFN)‐β

Toll‐like receptors include 10 and 13 family member proteins in humans and mice, respectively. The TLR family is evolutionally conserved and contains the ligand‐binding domains via leucine‐rich repeat (LRR) motifs at the N‐terminus and intracellular Toll/IL‐1 receptor (TIR) domain at the C‐terminus (Figure 1). TLRs act as the *gatekeepers* of host defense to various pathogens through structure‐ and sequence‐dependent immune recognition (Figure 1).^23^, ^24^, ^25^, ^26^ Most TLRs in humans and mice recognize similar PAMPs with some exceptions. TLR11, TLR12, and TLR13 have been lost in the human genome, and the *Tlr10* gene is disrupted in the mouse genome. TLR1 through to TLR9 are conserved in both species. TLR4 recognizes bacterial LPS. Triacyl and diacyl lipopeptides are recognized by dimerization of TLR2 with TLR1 and TLR6, respectively. TLR5 recognizes bacterial flagellin derived from flagella. TLR9 is a deoxyribonucleic acid (DNA) sensor and recognizes nonmethylated cytosine‐phosphate‐guanine (CpG) DNA. TLR3 recognizes double‐stranded RNAs (dsRNAs), and both TLR7 and TLR8 detect unmodified uridine‐rich single‐stranded RNAs (ssRNAs). TLRs localize either on the cell surface (TLR1, TLR2, TLR4, TLR5, and TLR6) or in endosomes (TLR3, TLR7, TLR8, and TLR9) through the transmembrane (TM) domain (Figure 1).

### 3.2 TLR signaling

Once activated by their ligands, individual TLRs recruit two major TIR‐containing adaptor molecules, myeloid differentiation primary response 88 (MyD88) and TIR‐domain‐containing adaptor inducing interferon‐β (IFN‐β) (TRIF) (Figure 1). The engagement of all TLRs except for TLR3 induces the signaling complex, named the Myddosome, which consists of MyD88 and IL‐1 receptor‐associated kinases (IRAKs), leading to activation of NF‐κB.^27^ The ligand engagement to TLR3 or TLR4 recruits TRIF, which results in activation of IFN regulatory factor 3 (IRF‐3) through a signalosome complex (TNF receptor‐associated factor 3 [TRAF3], TRAF‐associated NF‐κB activator [TANK]‐binding kinase 1 [TBK1], and inducible inhibitor of nuclear factor [NF]‐κB [IκB] kinase [IKKi], also known as IKKε, *IKBKE*). Phosphorylated IRF‐3 translocates into the nucleus and eventually induces the production of type I IFN.

### 3.3 TLR and allergy

Allergic development is believed to result from genetic backgrounds and environmental factors. Mutations in *TLR* family genes have been investigated using single nucleotide polymorphism (SNP) analysis and meta‐genome‐wide association studies (GWAS). Indeed, SNPs in the *TLR4* gene are a risk factor for asthma,^28^, ^29^ indicating that genetic variations of *TLR* family genes are related to susceptibility to allergic diseases.

Toll‐like receptor family proteins are differentially expressed in all cells types including macrophages, DCs, B cells, regulatory T (Treg) cells, and epithelial cells. They are directly capable of interacting with pathogens or foreign particles in the epithelial barrier and influence host immune cell responses with environmental factors. Barrier epithelial cells function as an origin of allergic response to external signals from the mucous membranes of the respiratory tract, intestinal tract, or skin. Tight junction barriers are extremely sensitive to detergents.^30^ LPS also increases tight junction permeability in a TLR4‐dependent manner.^31^ The dysregulation of the epithelial barrier may increase uptake of allergens in the pathogenesis of allergy.

Microbial‐treated TLR2/3/4/7/9 knockout mother mice are no longer protected from the development of asthma in their offspring,^5^ suggesting that maternal TLR signaling plays a pivotal role in the transfer of protective effects. However, the precise roles of TLRs in the development of allergic diseases are greatly influenced by many factors, such as cell types, expression level, and the nature of antigens. Indeed, TLR4 signaling leads to allergic responses.^32^, ^33^, ^34^ This TLR4‐mediated allergic reaction develops only by intranasal sensitization but not by subcutaneous or intraperitoneal sensitization, suggesting that TLR‐mediated reactions influence the dose of immune‐stimulatory components, as well as the route of administration and the timing of exposure. Treg cells have also been implicated in allergy development.^35^ Manipulation of Th1/Th2 balance or Treg cell function by administrating TLR agonists may be promising for the treatment of allergic diseases.^36^

### 3.4 Allergen‐specific immunotherapy for TLRs

Different TLR agonists have been assayed in clinical trials as adjuvants.^37^ These were further developed in the context of allergen‐specific immunotherapy (AIT) with different outcomes. Oral administration of TLR9 agonists displayed a significant benefit in the treatment of asthma and food allergy in mice.^38^ Several kinds of TLR agonists have now been applied to asthmatic or allergic patients in clinical trials.^37^, ^39^ In the case of TLR9 agonists, CpG‐containing nucleotides (Amb a 1‐CpG vaccine)^40^, ^41^ and bacteriophage‐derived virion‐like particles (VLPs) packaging A‐type CpG motif^42^ have been shown to be effective in treating rhinitis and allergic asthma. Intranasal administration of a TLR7 agonist (AZD8848) and TLR8 (VTX‐1463) has also reduced nasal symptoms in patients with allergic rhinitis.^43^ TLR4 agonist monophosphoryl A (MPL) promotes Th1 and Treg cell responses in cooperation with switching from IgE to IgG blocking antibody production.^39^

## 4 C‐TYPE LECTIN RECEPTORS

**Figure 2:**
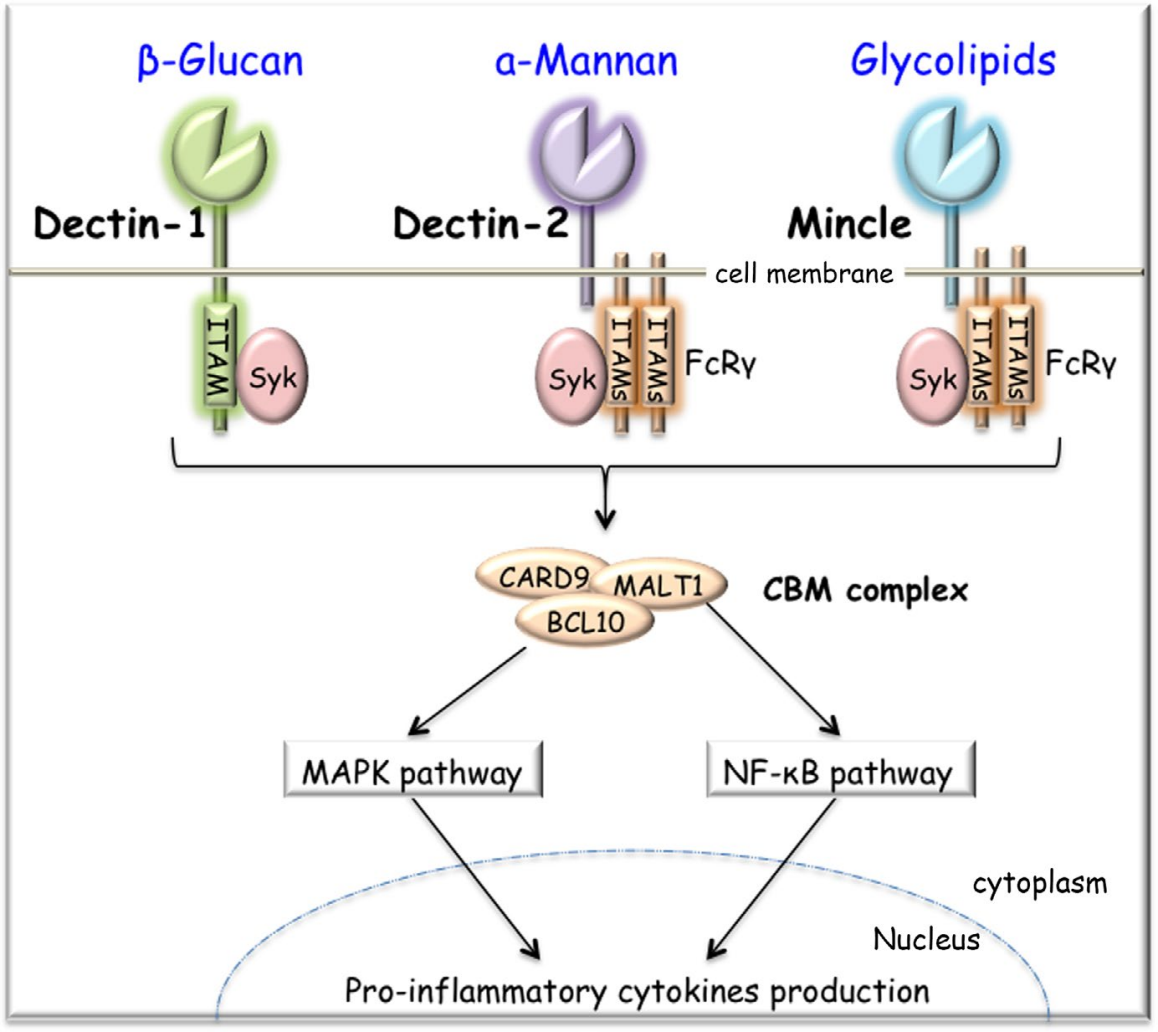
Type II transmembrane CLR. Type II transmembrane CLR proteins possess a single CRD. Dectin‐1 (*CLEC7A*) recognizes fungal wall‐derived β‐glucans. Dectin‐2 (*CLEC6A*) recognizes the structure of α‐mannans. Mincle (*CLEC4E*) recognizes diverse glycolipids including TDM, Glc‐DAG, and MGDG. Dectin‐1 transduces the signal through its ITAM‐like motif. Both Dectin‐2 and Mincle associate with FcRγ for signaling. Ligand‐bound CLRs result in the Syk‐dependent formation of the CBM (CARD9‐BCL10‐MALT1) complex. The CBM complex activates the pathways of MAPK and NF‐κB, leading to pro‐inflammatory cytokine production. BCL10, B‐cell CLL/lymphoma 10; CARD9, caspase‐recruitment domain 9; CLR, C‐type lectin receptor; CRD, carbohydrate recognition domain; Dectin‐1, dendritic cell (DC)‐associated C‐type lectin‐1; Dectin‐2, DC‐associated C‐type lectin‐2; FcRγ, Fc receptor common gamma chain; Glc‐DAG, glucosyl diacylglycerol; ITAM, immunoreceptor tyrosine‐based activation motif; MALT1, mucosa‐associated lymphoid tissue protein 1; MAPK, mitogen‐activated protein kinase; MGDG, monoglucosyldiacylglycerol; Mincle, macrophage‐inducible C‐type lectin; NF‐κB, nuclear factor‐kappa B; Syk, spleen tyrosine kinase; TDM, trehalose‐6,6′‐dimycolate

CLRs recognize a diverse range of nonself PAMPs derived from microbes, especially fungi and house dust mites.^44^, ^45^, ^46^ Most cells, including DCs and macrophages, express CLRs. CLRs belong to the C‐type lectin‐like domain (CTLD) superfamily, which carries the carbohydrate recognition domain (CRD). CLRs contain one or more conserved CTLD.^47^ Type II transmembrane CLRs, which possess a single CRD, have been most extensively studied among CLRs (Figure 2). This subfamily includes DC‐associated C‐type lectin‐1 (Dectin‐1, *CLEX7A*), Dectin‐2 (*CLEC6A*), macrophage‐inducible C‐type lectin (Mincle, *CLEC4E*), DC‐specific intracellular adhesion molecule 3 (ICAM3)‐grabbing nonintegrin (DC‐SIGN*, CD209*), and DC NK lectin group receptor‐1 (DNGR‐1, *CLEC9A*).

### 4.1 Dectin‐1, Dectin‐2, and Mincle

Dectin‐1 and Dectin‐2 recognize fungal wall‐derived β‐glucan and α‐mannan structure, respectively^48^ (Figure 2). Both are organized in the gene cluster in the human and mouse genomes.^49^, ^50^ Mincle, a member of the Dectin‐2 family, recognizes various glycolipids (Figure 2), such as trehalose‐6,6′‐dimycolate (TDM) in the cell wall of *Mycobacterium tuberculosis*,^51^ glucosyl diacylglycerol (Glc‐DAG) of *Streptococcus pneumoniae*,^52^ monoglucosyldiacylglycerol (MGDG) produced by Group A *Streptococcus*,^53^ and others derived from self and nonself.^54^

### 4.2 CLR signaling

Dectin‐1 directly transduces the signal through its immunoreceptor tyrosine‐based activation motif (ITAM)‐like motif in the cytoplasmic domain. Dectin‐2 and Mincle are required for the ITAM‐containing adaptor protein Fc receptor common gamma chain (FcRγ, *FCER1G*) (Figure 2). Once ligands are bound to CLRs in a calcium‐dependent manner, spleen tyrosine kinase (Syk) is recruited to phosphorylated ITAM motifs, leading to cellular activation. In this signaling cascade, caspase‐recruitment domain (CARD)‐containing adaptor proteins, CARD9 and B‐cell CLL/lymphoma 10 (BCL10), form a complex with caspase‐like cysteine protease mucosa‐associated lymphoid tissue protein 1 (MALT1). CARD9‐BCL10‐MALT1 (CBM) complex activates NF‐κB and mitogen‐activated protein kinase (MAPK) pathways, resulting in the production of pro‐inflammatory cytokines (Figure 2).

### 4.3 CLRs and allergy

Dectin‐1 is involved in fungal‐mediated allergic inflammation mediating T‐helper 17 (Th17) cell differentiation.^55^, ^56^ Genetic polymorphisms of Dectin‐1, TLR3, and TLR9 are significantly associated with susceptibility to severe asthma with fungal sensitization.^57^ Dectin‐2 has been implicated in allergic inflammation to house dust mites with Th2 polarization.^58^, ^59^, ^60^ A recent study shows that Mincle recognizes not only glycolipids but also self‐derived cholesterol sulfate in skin epithelial cells and is involved in the induction of allergic skin inflammatory response.^61^

### 4.4 Allergen‐specific immunotherapy for CLRs

Recent findings have shown that allergoids conjugated to nonoxidized mannan from *Saccharomyces cerevisiae* are next‐generation vaccines targeting DCs through CLRs. These vaccines are candidates for AIT of allergic diseases as they promote the generation of Treg cells by mechanisms partially depending on programmed death‐ligand 1 (PD‐1) and IL‐10 in both humans and mice.^62^, ^63^ Phase II clinical trials for grass‐pollen and house dust mite allergens are currently ongoing.^64^

## 5 RIG‐I‐LIKE RECEPTORS

### 5.1 RLR family

**Figure 3:**
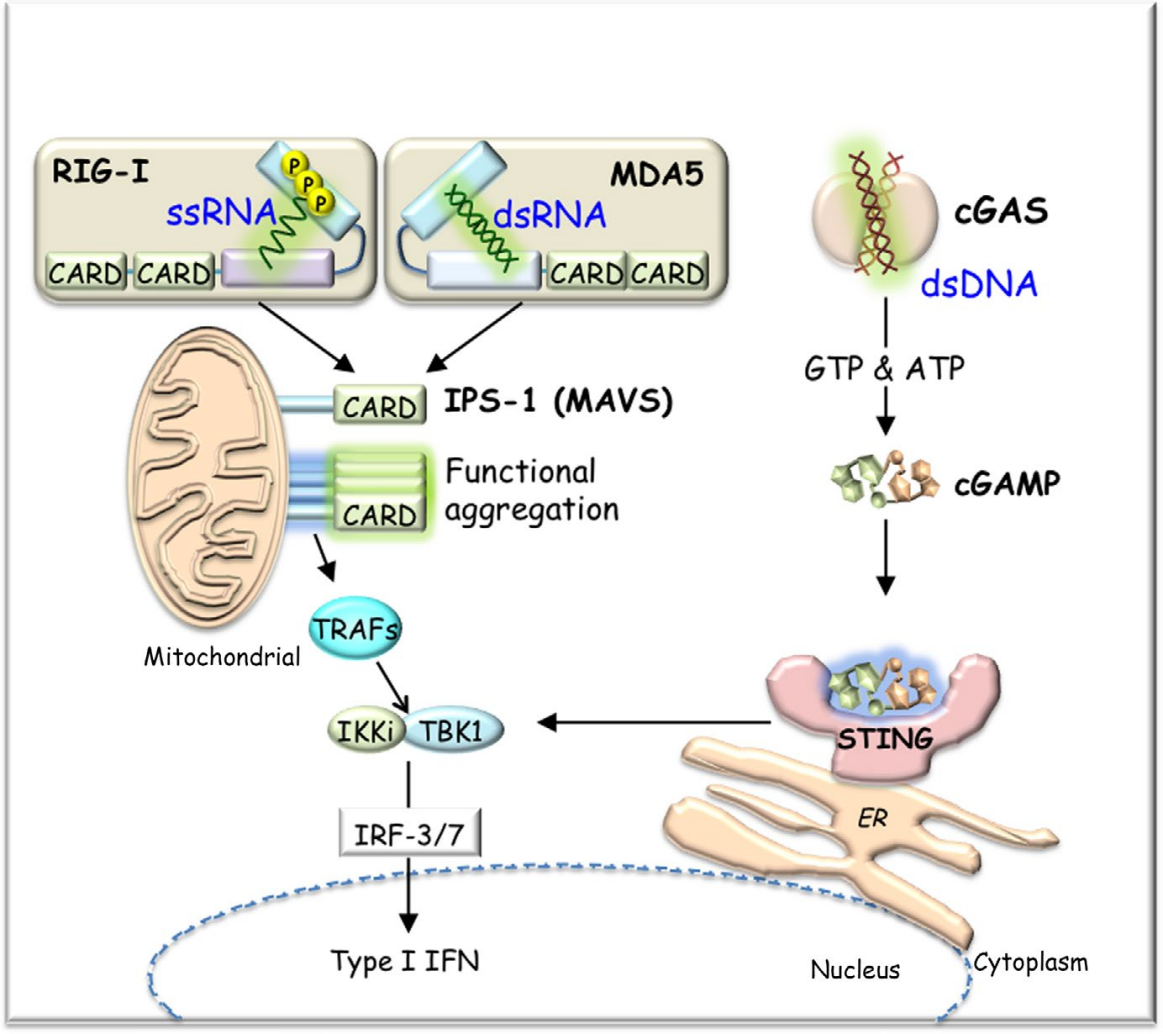
Nucleic acid sensors. RIG‐I and MDA5 have two CARD domains at the N‐terminus and a helicase domain at the center. Both RIG‐I and MDA5 bind viral RNAs bearing 5’‐triphosphate or 5’‐diphosphate distinct from the mammalian RNA with 5’ cap structure. After viral RNA recognition, RIG‐I and MDA5 interact with IPS‐1 (MAVS) through CARD‐CARD interactions. IPS‐1 (MAVS) is localized on the mitochondrial outer membrane. Aggregated IPS‐1 (MAVS) activates TBK1 and IKKi (IKKε) through TRAFs, leading to induction of type I IFN via phosphorylation of IRF‐3/7. cGAS binds dsDNA and produces a second messenger, cGAMP, from ATP and GTP. cGAMP binds to STING localized on the ER membrane and induces type I IFN production via the TBK1/IRF‐3 pathway. CARD, caspase‐recruitment domain; cGAMP, cyclic GMP‐AMP; cGAS, cyclic GMP‐AMP synthase; ER, endoplasmic reticulum; IKKi, inducible inhibitor of NF‐κB (IκB) kinase; IPS‐1, *IFN‐β* promoter stimulator 1; IRF, IFN regulatory factor; MDA5, melanoma differentiation‐associated gene 5; RIG‐I, retinoic acid‐inducible gene I; STING, stimulator of IFN genes; TBK1, TRAF‐associated NF‐κB activator (TANK)‐binding kinase 1; TRAF, TNF receptor‐associated factor

*Tlr3* ‐deficient cells showed normal type I IFN production toward viral infection,^65^ suggesting that additional mechanisms were hidden in the RNA sensing. As the cytosolic dsRNA sensor sensing both RNA helicases, RIG‐I (*DDX58*)^66^ and melanoma differentiation‐associated gene 5 (MDA5, *IFIH1*) 67 were identified.^68^ The RLR family proteins possess the DExHD motif containing the helicase domain for dsRNA recognition and have the two CARD domains at the N‐terminus (Figure 3).^69^ Laboratory of genetics and physiology 2 (LGP2, *DHX58*) lacks a CARD domain and, therefore, has no intrinsic signaling activity.

### 5.2 RLR recognition and signaling

Retinoic acid‐inducible gene I and MDA5 have different roles in the recognition of RNA viruses.^70^ RIG‐I recognizes relatively short viral RNA blunt ends bearing 5′‐di‐/triphosphate distinct from the host cellular RNA with 5′ cap structure.^71^, ^72^, ^73^ RIG‐I detects many RNA viruses, such as rhinovirus, Sendai virus, vesicular stomatitis virus, and influenza virus. RIG‐I‐mediated RNA recognition mechanisms are viral replication‐independent.^71^ MDA5 responds to longer (over 1k bp) dsRNA,^74^ such as polyinosinic‐polycytidylic acid [poly(I:C)], as well as dsRNA generated after infection of picornaviruses including encephalomyocarditis virus (EMCV), Mengo virus, and Theiler's virus. After viral RNA recognition, RIG‐I and MDA5 interact with the downstream CARD‐containing adaptor protein, *IFN‐β* promoter stimulator 1 (IPS‐1, *MAVS*), through CARD‐CARD interactions^75^, ^76^, ^77^, ^78^ (Figure 3). IPS‐1 localizes on the mitochondrial outer membrane, which triggers prion‐like aggregation of IPS‐1^79^ (Figure 3). The aggregated IPS‐1 recruits IRF‐3 kinase and activates the IRF‐3‐TBK1‐IKKi (IKKε)‐IRF‐3/7‐IFN‐dependent signaling pathway through TRAFs.

### 5.3 RLRs and allergy

Early viral infections in children are associated with further allergic sensitization and asthma persistence. Similarly, viral infections in asthma patients (both allergic and nonallergic) are also associated with asthma exacerbations. In the development of respiratory disease, innate immune mechanisms are involved in virus‐infected airway epithelial cells.^80^ Loss‐of‐function mutations in the *IFIH1* gene increase susceptibility to severe respiratory infection caused by human rhinovirus in children.^81^, ^82^ A meta‐phenome‐wide association study also revealed a novel association of an *IFIH1* allele mutation to increased risk for asthma.^83^ In an experimental model, poly(I:C) and rhinovirus‐derived dsRNA exacerbated asthma.^84^, ^85^, ^86^ Taken together, RLRs play a nonredundant and critical role in the development and progression of asthma.

### 5.4 cGAS—DNA sensor

Recent studies have revealed the existence of a new intracellular DNA sensing system. Cyclic GMP‐AMP synthase (cGAS), a member of nucleotidyltransferase family, binds dsDNA in a sequence‐independent manner but is activated in a length‐dependent manner (longer than 94‐bp DNA).^87^ cGAS undergoes a conformational change of its catalytic center and then produces the cyclic GMP‐AMP (cGAMP) from ATP and GTP (Figure 3).^88^, ^89^, ^90^ cGAMP acts as second messenger,^91^, ^92^ binds to the stimulator of IFN genes (STING),^93^, ^94^, ^95^ localizes on the endoplasmic reticulum (ER) membrane, and induces type I IFN production via the TBK1/IRF‐3 pathway (Figure 3).^96^, ^97^ cGAMP senses viral‐derived DNA as well as host‐derived DNA.^98^, ^99^ cGAS is involved in DNA damage‐induced inflammatory signaling in cancer cells.^100^, ^101^ The cGAS‐STING‐mediated DNA sensing system also contributes to the induction of apoptosis, control of ER stress response, and autophagy.

## 6 NOD‐LIKE RECEPTORS

**Figure 4:**
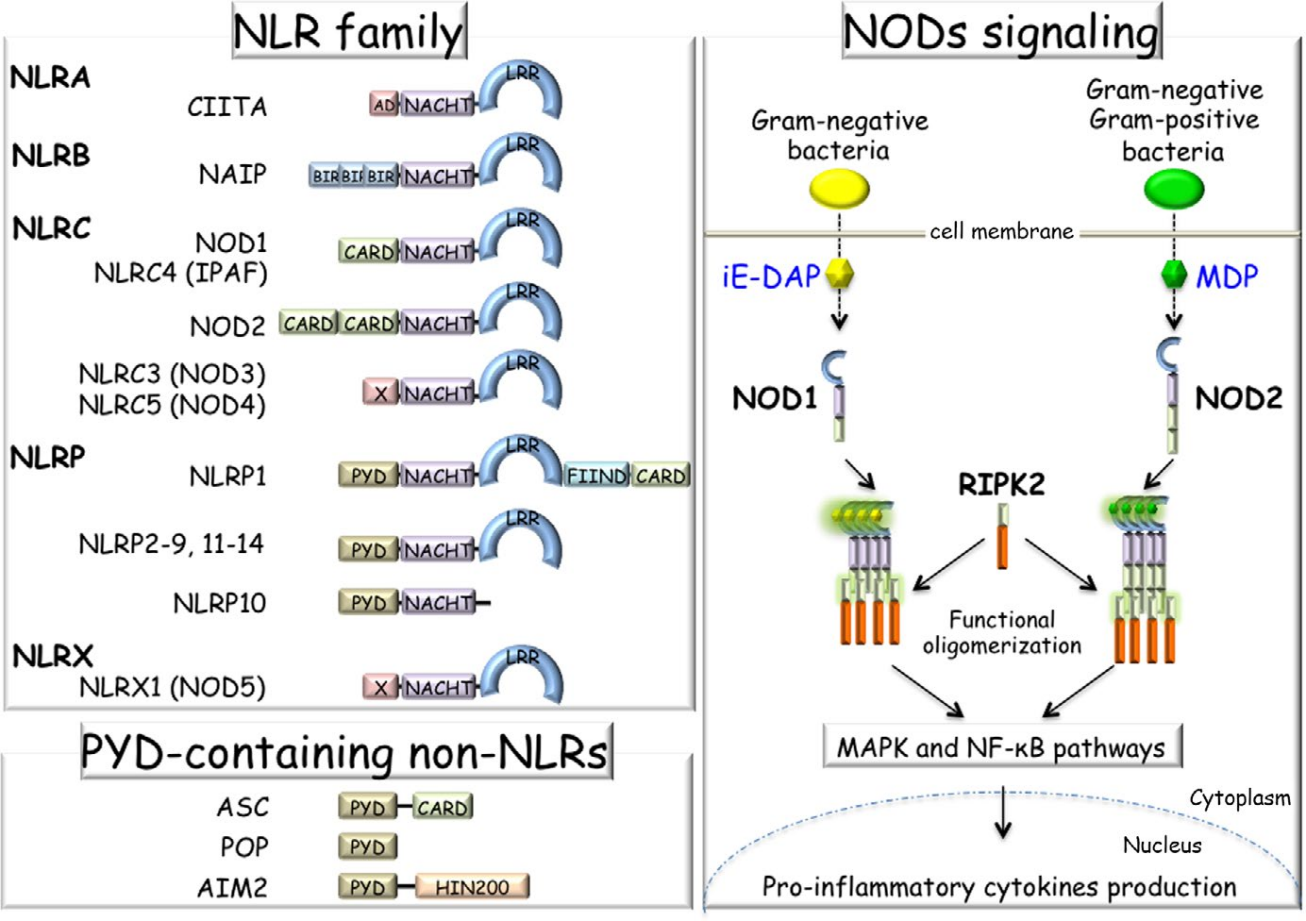
NLR family, PYD‐containing non‐NLR proteins, and NOD signaling. In the NLR family, there are five major subfamilies based on the unique N‐terminal domain structure: NLRA (AD‐type), NLRB (BIR‐type), NLRC (CARD‐type), NLRP (PYD‐type) and NLRX (X‐type). All family proteins have a NACHT domain in the central region. NOD2 senses bacterial cell wall‐derived peptidoglycan derivative MDP; however, NOD1 only senses Gram‐negative bacteria‐derived iE‐DAP. After sensing ligands, NODs oligomerize and interact with RIPK2 through the CARD domain. RIPK2 further activates downstream cascades and MAPK and NF‐κB pathways, leading to cytokine production. AD, transactivation domain; BIR, baculoviral inhibitor of apoptosis repeat; CARD, caspase‐recruitment domain; FIIND, function‐to‐find domain; HIN200, hematopoietic interferon‐inducible nuclear antigens with 200 amino acid repeats; iE‐DAP, γ‐D‐glutamyl‐meso‐diaminopimelic acid; LRR, leucine‐rich repeat; MAPK, mitogen‐activated protein kinase; MDP, muramyl dipeptide; NACHT, NAIP, CIIA, HeT‐E, and TEP1; NF‐κB, nuclear factor‐kappa B; NOD1, nucleotide‐binding oligomerization domain‐containing protein 1; NOD2, nucleotide‐binding oligomerization domain‐containing protein 2; PYD, Pyrin domain; RIPK2, receptor‐interacting serine/threonine‐protein kinase 2

A third group of PRRs are NLRs.^102^ NLRs are localized in the cytosol and recognize PAMPs and DAMPs. NLRs carry three unique structural properties (Figure 4); the central region is named NOD or nucleotide‐binding domain (NACHT), which consists of conserved motifs including ATP/GTPase‐specific phosphate‐binding loop and magnesium‐binding site; the C‐terminal region contains LRRs, which respond to ligand specificity similar to TLRs; the N‐terminal region is different among NLRs (Figure 4). Based on the name, NLRs are divided into five major subgroups (NLRA, NLRB, NLRC, NLRP, and NLRX) (Figure 4). So far, twenty‐five NLR family genes have been identified in the human genome.

### 6.1 NOD1 and NOD2

NOD1 (*CARD4*) and NOD2 (*CARD15*) are founding members of NLR family^103^ and belong to the NLRC subfamily. NOD1 has a single CARD domain and NOD2 has two CARD domains at the N‐terminus (Figure 4). Both NOD1 and NOD2 sense bacterial cell wall‐derived peptidoglycan derivative γ‐D‐glutamyl‐meso‐diaminopimelic acid (iE‐DAP) and muramyl dipeptide (MDP) structures, respectively (Figure 4). iE‐DAP is derived from Gram‐negative bacteria, whereas MDP is derived both from Gram‐positive and Gram‐negative strains.

### 6.2 NOD signaling

Upon recognition, NODs oligomerize and interact with CARD‐containing receptor‐interacting serine/threonine‐protein kinase 2 (RIPK2) through the CARD domain, and then activate transforming growth factor (TGF)‐β‐activated kinase 1 (TAK1, *MAP3K7*) and IKK complex for leading to MAPK and NF‐κB activation^104^ (Figure 4). NOD1 is widely expressed by a variety of cell types, whereas NOD2 expression is limited to certain cell types such as hematopoietic cells^105^ and Paneth cells in the intestine.^106^

### 6.3 NODs and allergy

NOD1 and NOD2 promote Th1 and Th17 adaptive immunity by inducing the secretion of TNF and IL‐1^107^, ^108^ in addition to Th2 immune response,^109^ suggesting that signaling through these receptors may be central to susceptibility and exacerbation of allergies. Although inhalation of NOD1 and NOD2 ligands induces Th2 response, NOD2 appears to have more potent activity than NOD1 in Th2‐driven allergic inflammation. It is shown that NOD2 displays this function by promoting the expression of TSLP, OX40 ligand (OX40L/CD252, *TNFSF4*), and IL‐25.^110^ NOD2‐induced suppression of Treg cell development and induction of early IL‐4‐secreting cells are completely dependent on TSLP. NOD1 cannot induce strong TSLP expression. However, intranasal infusion of high doses of NOD2 ligands did not break tolerance nor lead to asthma susceptibility, indicating a dose‐dependent effect of NOD2 in allergy development.

Polymorphisms of NOD1 and NOD2 are highly associated with inflammation development in the respiratory airways,^111^ childhood asthma,^112^ and atopic diseases.^113^, ^114^, ^115^ It has been thought that these phenotypes arise from a defect in NOD sensing fragments of bacterial peptidoglycan. However, a recent study shows that NODs also participate in sensing infection with viruses and parasites by inducing ER stress‐induced inflammation,^116^ and further research is needed to elucidate the role of NODs in allergic diseases.

### 6.4 PYD‐containing non‐NLRs

The NLRP family has the Pyrin domain (PYD) at the N‐terminal region. Similarly to the CARD domain, PYD prefers to assemble together; thus, PYD‐containing proteins give rise to a complex. The complex formation of NLRs is important for signaling and inflammation. The overall composition of NLRs is not well characterized yet, but it is important to uncover the role of NLRs, especially in the inflammasome (Section 7). Studies on additional small molecules, such as PYD‐only proteins (POPs) family (Figure 4) and CARD‐only proteins (COPs) family, may provide a more detailed mechanism.^117^, ^118^, ^119^

## 7 INFLAMMASOME

**Figure 5:**
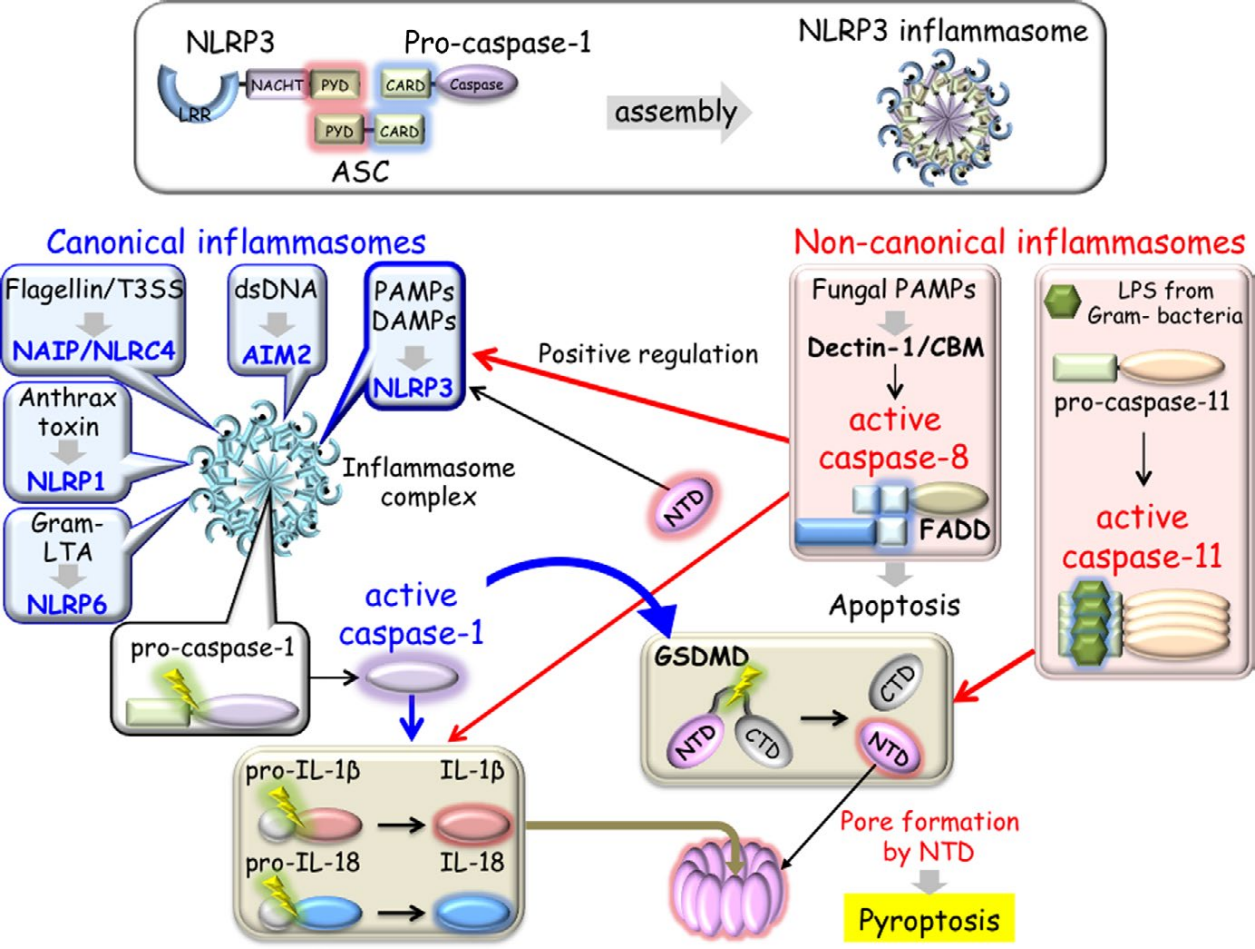
Inflammasome. NLRP3 forms a complex with ASC through their PYD binding each other and recruiting the CARD domain of pro‐caspase‐1. After assembly, this multimeric complex is called the inflammasome (upper box). In the canonical inflammasome assembly, ligand‐sensing NLRs (NLRP1, NLRP3, NLRP6, NAIP/NLRC4, and AIM2) (blue balloons) form multisubunit disk‐like structures comprising an inner ring and outer ring. Pro‐caspase‐1 is located in the central portion (black balloon). Activated caspase‐1 processes IL‐1β/IL‐18 and triggers proteolytic cleavage of GSDMD (brown boxes). In the noncanonical inflammasomes, caspase‐11 and caspase‐8 participate in the cytoplasmic LPS sensing pathway and Dectin‐1‐CBM signaling pathway, respectively (pink boxes). Activated caspase‐11 cleaves GSDMD similarly to caspase‐1 (red arrow). GSDMD‐derived NTD induces pore formation for pyroptosis and stimulates the NLRP3 inflammasome pathway (black arrows). Activated caspase‐8 with FADD is capable of cleaving pro‐IL‐1β/pro‐IL‐18 (red arrow). Caspase‐8 may positively regulate NLRP3 inflammasome pathway (red arrow). AIM2, absent in melanoma 2; ASC, apoptosis‐associated speck‐like protein containing a CARD; CARD, caspase‐recruitment domain; CBM, CARD9‐BCL10‐MALT1; Dectin‐1, lipopolysaccharide; GSDMD, gasdermin D; LPS, lipopolysaccharide; LTA, lipoteichoic acid; NAIP, NLR family apoptosis inhibitory protein; NLRC4, NLR family CARD domain containing 4; NLRP1, NLR family Pyrin domain containing 3; NLRP3, NLR family Pyrin domain containing 3; NLRP6, NLR family Pyrin domain containing 6; NTD, N‐terminal domain; PYD, Pyrin domain

NLRP family proteins, a subgroup of NLR family, and absent in melanoma 2 (AIM2) form a complex with apoptosis‐associated speck‐like protein containing a CARD (ASC, *PYCARD*) through their PYD domains and recruit the CARD domain of pro‐caspase‐1, forming the inflammasome^120^ (Figure 5). Inflammasomes are cytosolic multimeric protein complexes sensing and responding to pathogenic microbes and cellular damage.^121^ To date, various inflammasomes including NLR subfamily proteins (NLRP1 [*CARD7*], NLRP2, NLRP3, NLRP4, NLRP6, and NLRC4 [*CARD12*]) and PYD‐containing non‐NLR proteins (AIM2, pyrin (*MEFV*), and IFN‐γ‐inducible protein (IFI) 16) have been identified. Based on the activation mechanism, the inflammasomes are categorized as canonical or noncanonical.

### 7.1 Canonical inflammasome

Caspases are a group of cysteine‐aspartic acid proteases, of which 12 and 10 family members have been identified in humans and mice, respectively. Caspases play an essential role in programmed cell death and are involved in either apoptosis or inflammation along with pyroptosis^122^ (Section 8). In the steady state, the caspase family is present in inactive forms called zymogens, which require activation. Inflammasome mediates the activation of caspase‐1 through CARD‐CARD interactions. Caspase‐1, caspase‐4 (human only), caspase‐5 (human only), caspase‐11 (mouse only), and caspase‐12 are known to be involved in the inflammatory pathway, and those inflammatory caspase genes are clustered in the human and mouse genome.^123^ Inflammasomes activate caspase‐1, resulting in production of pro‐inflammatory cytokines IL‐1β and IL‐18 upon activation by various signals^124^ (Figure 5). Thus, “canonical” inflammasome relies on the activation of caspase‐1.

#### 7.1.1 NLRP3 inflammasome

NLRP3 inflammasomes can respond to a variety of substances including crystals such as urate, cholesterol, asbestos, silica, aggregated amyloid‐β, and islet amyloid polypeptide.^125^ As a direct interaction of NLRP3 with these substances has not been detected, NLRP3 inflammasome activation is considered to occur via mitochondrial and lysosomal damage. Reactive oxygen species (ROS) also trigger NLRP3 inflammasomes priming.^126^ Extracellular ATPs released from activated or necrotic cells activate P2X purinoceptor 7 (P2X7, *P2RX7*) receptor^127^ and induce caspase‐1 activation, leading to IL‐1β production via formation of an NLRP3 inflammasome.^128^ IL‐1β is critically involved in Th17 cell differentiation, production of Th17‐associated cytokines, and neutrophilic inflammation.^129^ In addition to the Th2 response, a Th17 response is associated with asthma, notably severe glucocorticoid‐resistant asthma.^130^ From various studies of human diseases and mouse models, the elevation of IL‐1β and IL‐18 is associated with the development of allergic diseases, such as asthma, dermatitis, rhinitis, and conjunctivitis.^131^ All these events are connected with inflammasome activation and suggest participation of the inflammasome in the development and progression of asthma. Th2 and Th17 inflammatory pathways are mutually regulated in asthmatic patient samples.^132^ Type II cytokine suppression promotes Th17 responses, indicating that combined therapy targeting both Th2 and Th17 responses may benefit asthmatic patients.

#### 7.1.2 AIM2 inflammasome

Cytoplasmic dsDNA sensor AIM2 is a member of IFI20X/IFI16 family.^133^ In normal conditions, the N‐terminal PYD of AIM2 maintains an autoinhibitory state through interaction with C‐terminal hematopoietic IFN‐inducible nuclear proteins with a 200‐amino acid repeat (HIN‐200) domain, which directly binds to the sugar‐phosphate backbone of dsDNA in a sequence‐independent manner.^134^, ^135^ The ligand binding to the HIN‐200 domain triggers the activation of the AIM2 inflammasomes, leading to pyroptosis and the production of IL‐1β and IL‐18.^136^, ^137^, ^138^, ^139^ The AIM2 inflammasomes play an important role in infection, cancer, and autoimmunity 140 by sensing of virus/bacteria‐derived dsDNA and tumor‐derived DNA.^141^, ^142^ A recent study shows that AIM2 is dispensable for inflammasome activation in some primary human myeloid cells, where STING‐dependent cell death can trigger NLRP3 inflammasome activation by inducing potassium efflux.^143^ This result implicates the presence of a cell‐type‐specific alternative pathway in the dsDNA sensing system. Inflammasome‐derived caspase‐1 binds to cGAS and restricts its downstream STING‐TBK1‐IRF‐3/7‐IFN‐dependent signaling pathway by cutting out cGAS,^142^ implicating a crosstalk between type I IFN production and the inflammasome. cGAMP also functions in the priming and activation of AIM2 and NLRP3 inflammasomes.^144^

#### 7.1.3 Other inflammasomes

NLRC4 inflammasomes are activated by NLR family apoptosis inhibitory protein (NAIP) harboring three baculoviral inhibitor of apoptosis repeat (BIR) domains at N‐terminus.^145^, ^146^ NAIP family proteins detect monomeric flagellin or the needle/rod regions of type III secretion system (T3SS),^147^, ^148^, ^149^, ^150^ leading to the activation of downstream NAIP/NLRC4 inflammasome responses. The transcription of *Naips* is regulated by the transcription factor IRF‐8.^151^ NLRC4 is implicated in the exacerbation of psoriatic lesions,^152^ and NLRP4 mutation is associated with exacerbation of asthma in smoking adults.^153^ Polymorphisms of NACHT‐LRR in NLRP12 and the promoter regions of NLRC4 and NLRP1 are associated with atopic dermatitis.^113^ The elusive other inflammasomes remain of outstanding interest.

### 7.2 Noncanonical inflammasome

Caspase‐11 senses cytosolic LPS with noncanonical function^154^, ^155^, ^156^, ^157^, ^158^ (Figure 5). This intracellular LPS sensing can trigger caspase‐11‐mediated pyroptosis under a TLR4‐independent mechanism.^159^, ^160^, ^161^ In this caspase‐1‐independent pathway, murine caspase‐11 (caspase‐4 and caspase‐5 in humans) directly senses LPS through its CARD domain.^162^ Thus, “noncanonical” inflammasomes can lead to the activation of caspase‐11. The NLRP3 inflammasome is involved in both canonical and noncanonical activation. Interestingly, the NLRP6 inflammasome senses Gram‐positive bacteria‐derived lipoteichoic acid (LTA) (Figure 5).^163^ In this activation mechanism, NLRP6 recruits both caspase‐1 and caspase‐11. The processed caspase‐11 induces caspase‐1 activation, resulting in the production of IL‐1β and IL‐18.

#### 7.2.1 Caspase‐8

Caspase‐8 is known to play a central role in apoptosis as initiator and apical activator. Caspase‐8 has two death effecter domains (DEDs) in its N‐terminus and is structurally different to CARD‐containing inflammatory caspases. Interestingly, caspase‐8 has been shown to form a noncanonical inflammasome in response to fungal and mycobacterial infection by Dectin‐1.^164^ Fungal PAMPs activate Dectin‐1 signaling to induce a noncanonical caspase‐8‐ASC with CBM complex in a caspase‐1‐independent manner.^165^ Caspase‐8 assumes its inflammatory roles by inducing IL‐1β activation^165^, ^166^ (Figure 5). Furthermore, caspase‐8 mediates both canonical and noncanonical NLRP3 inflammasome priming and activation with a death domain (DD)‐containing adaptor protein Fas‐associated protein with DD (FADD).^167^ Caspase‐8‐mediated IL‐1 signaling promotes Th2 responses in allergic airway inflammation,^168^ implicating its therapeutic potential for asthma.

### 7.3 Inhibition of inflammasome activation

Inflammasomes are multiple protein complexes; therefore, abnormal assembly causes hyperinflammatory conditions, as in the case of skin inflammation in NLRP1 germline mutation.^169^ Some pathogens are able to selectively inhibit the activation of the caspase‐11‐mediated noncanonical NLRP3 inflammasome.^170^ However, the regulatory mechanisms of inflammasomes are not fully understood and need further characterization. Interestingly, a recent study shows that TAK1 restricts the NLRP3 inflammasome to regulate cell homeostasis and death in myeloid cells.^171^ *TAK* deficiency promotes spontaneous NLRP3 inflammasome activation. TAK1 inhibits the activation of DD‐containing receptor‐interacting serine/threonine‐protein kinase 1 (RIPK1). Activated RIPK1 induces the caspase‐8‐FADD pathway. TAK1 inactivation induces RIPK1 activation, leading to the caspase‐8‐dependent pathway,^172^ indicating that RIPK1 plays a role upstream of caspase‐8. This machinery is also associated with neuroinflammation, aging, and infection.^173^ Hence, the mechanism requires further investigation to understand the implications of inhibiting inflammasome activation.

## 8 PYROPTOSIS

### 8.1 Pyroptosis

Pyroptosis is one form of cell death and is morphologically different to apoptosis and necrosis. Apoptosis is an immunologically silent death mode while necrosis and pyroptosis are pro‐inflammatory death modes tightly associated with inflammation.^174^ Caspase‐1 is a key player of pyroptosis in cell death (canonical inflammasome). As described above, caspase‐11 also contributes to the central mechanism of pyroptosis (Figure 5).^155^ Pyroptotic cells release their entire cellular contents including nuclear and mitochondrial DNA. Pyroptosis preferentially occurs in macrophages, monocytes, and DCs. Neutrophil cell death is called NETosis (neutrophil extracellular traps) and releases chromatin components to the extracellular space.^175^, ^176^ It is found that NETosis‐derived dsDNA mediates allergic asthma exacerbations during rhinovirus infection,^177^, ^178^ suggesting dsDNA acts as an adjuvant to boost type II‐mediated allergic inflammation.

### 8.2 Gasdermin

The gasdermin (GSDM) family consists of six member proteins (GSDMA, GSDMB, GSDMC, GSDMD, GSDME, and Pejvakin).^179^ The molecular mechanisms underlying pyroptosis and GSDM family functions have been elucidated.^161^, ^180^, ^181^, ^182^, ^183^, ^184^, ^185^, ^186^ Activated caspases directly cleave GSDMD into two fragments, the N‐terminal domain (NTD) and C‐terminal domain (CTD). The NTD of GSDMD oligomerizes to form a pore on the cell membrane. This formation perforates the plasma membrane and initiates pyroptosis, leading to inflammasome‐mediated secretion of mature IL‐1β and IL‐18 (Figure 5). Recent studies show that GSDMD is involved not only in pyroptosis but also in NETosis.^187^, ^188^, ^189^

### 8.3 GSDMB and allergy

Importantly, the genome locus of GSDMB and orosomucoid 1‐like 3 (ORMDL3) on chromosome 17q21 is strongly associated with childhood‐onset asthma.^190^, ^191^ GSDMB is highly expressed in airway epithelial cells.^192^ GSDMB‐mediated pyroptosis in epithelial cells may be involved in the pathogenesis of asthma. Furthermore, GSDMB transgenic mice assume asthma symptoms in the absence of airway inflammation,^192^ implicating that the induction of GSDMB triggers asthma.

## 9 ALPK1‐TIFA‐NF‐κB AXIS

**Figure 6:**
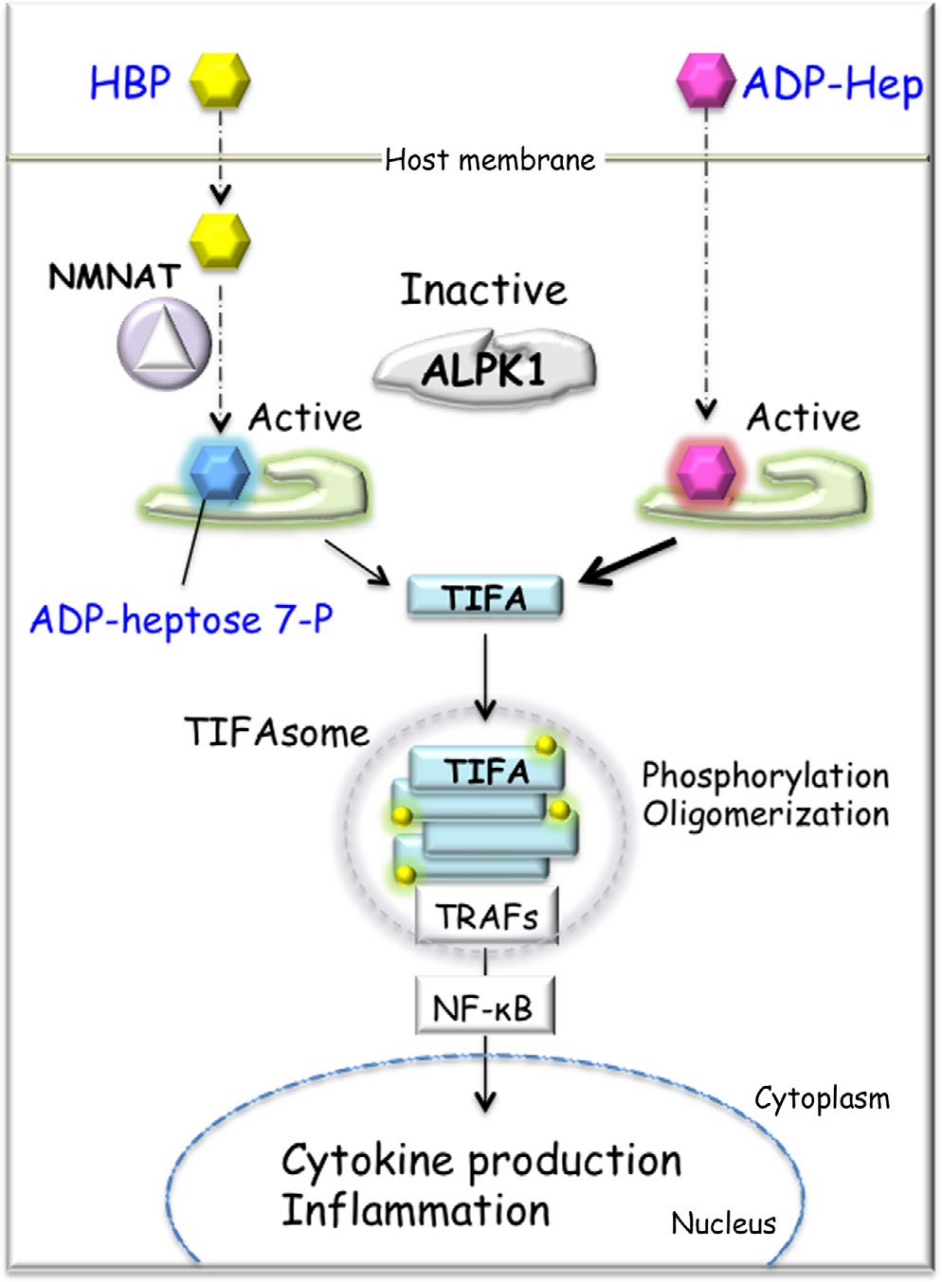
ALPK1‐TIFA‐NF‐κB axis. Once HBP and ADP‐Hep, bacterial products of the LPS biosynthetic pathway, are transported into the host cell, both types of sugars activate NF‐κB signaling pathway. ADP‐heptose 7‐P, which is converted from HBP by host enzyme NMNAT, interacts to N‐terminus of ALPK1. Activated ALPK1 phosphorylates TIFA and induces the TIFA oligomerization with TRAFs, named TIFAsome. ADP‐Hep can also interact with ALPK1 in the same fashion, leading to the activation of NF‐κB and inflammation. ADP‐Hep has much more potent NF‐κB activator than HBP. ADP‐Hep, ADP‐β‐D‐manno‐heptose; ALPK1, alpha‐kinase 1; HBP, D‐glycero‐β‐D‐manno‐heptose 1,7‐bisphosphate; LPS, lipopolysaccharide; NMNAT, nicotinamide mononucleotide adenylyltransferase; TIFA, TRAF‐interacting protein with forkhead‐associated domain; TRAF, TNF receptor‐associated factor

Besides LPS itself, ADP‐β‐D‐manno‐heptose (ADP‐Hep) and D‐glycero‐β‐D‐manno‐heptose 1,7‐bisphosphate (HBP),^193^, ^194^, ^195^ intermediate products of the LPS biosynthetic pathway, activate the NF‐κB signaling pathway (Figure 6). ADP‐Hep is more potent than HBP. TRAF‐interacting protein with forkhead‐associated domain (TIFA) was originally identified as a TRAF2‐binding protein that is involved in the NF‐κB pathway.^196^ Both ADP‐Hep and HBP sensing trigger TIFA oligomerization.^193^ Recent studies have shown that TIFA oligomerization can be induced by ADP‐Hep or ADP‐heptose 7‐P that is converted from HBP by host adenylyltransferase enzymes of the nicotinamide mononucleotide adenylyltransferase (NMNAT) family (Figure 6).^197^ Alpha‐kinase 1 (ALPK1), a member of the atypical kinase family alpha kinases, is necessary for phosphorylation‐dependent formation of TIFA oligomerization.^194^, ^195^ These sugar molecules directly bind the N‐terminal domain of ALPK1, stimulating its kinase domain to phosphorylate and activate TIFA. The role of ADP‐Hep as a PAMP was further confirmed on comparison with synthetic HBP.^198^ This ADP‐heptose sensing system stimulates host innate immune responses.

## 10 CONCLUSION

Over 20 years, we have witnessed a remarkable advance in understanding the mechanism of pathogen recognition by the innate immune system. New players involved in the innate immune system continue to be reported. However, despite enormous efforts, our knowledge of how the innate immune system is involved in the development of allergic diseases is still limited, and feasible target molecules or pathways are yet to be discovered. It is necessary to determine how pathogen recognition molecules and subsequent signaling pathways are involved in the development of allergic diseases.

## CONFLICTS OF INTEREST

Kazuhiko Maeda has research collaboration with Otsuka Pharmaceutical Co., Ltd. Shizuo Akira has research support from Chugai Pharmaceutical Co., Ltd. The terms of this arrangement have been reviewed and approved by Osaka University in accordance with its policy on objectivity in research.

## References

[1] Kawai T , Akira S . The roles of TLRs, RLRs and NLRs in pathogen recognition. Int Immunol. 2009;21:317‐337. 19246554 10.1093/intimm/dxp017 PMC2721684

[2] Janeway CA Jr , Medzhitov R . Constructing and contesting legitimacy and accountability in polycentric regulatory regimes. Annu Rev Immunol. 2008;20(2): 197‐216.

[3] Stefka AT , Feehley T , Tripathi P , et al. When institutions matter: union growth and decline in Western Europe, 1950‐1995. Eur Sociol Rev. 1999;15(2):135‐158.

[4] Lambrecht BN , Hammad H . The immunology of the allergy epidemic and the hygiene hypothesis. Nat Immunol. 2017;18:1076‐1083. 28926539 10.1038/ni.3829

[5] Conrad ML , Ferstl R , Teich R , et al. Maternal TLR signaling is required for prenatal asthma protection by the nonpathogenic microbe Acinetobacter lwoffii F78. J Exp Med *.* 2009;206:2869‐2877. 19995952 10.1084/jem.20090845 PMC2806458

[6] Fujimura KE , Lynch SV . Microbiota in allergy and asthma and the emerging relationship with the gut microbiome. Cell Host Microbe *.* 2015;17:592‐602. 25974301 10.1016/j.chom.2015.04.007 PMC4443817

[7] West CE , Renz H , Jenmalm MC , et al. Bacteriology in the service of sanitation: the factory environment and the regulation of industrial anthrax in late‐victorian Britain. Soc Hist Med. 2012;25:343‐361.

[8] Walton KL , Galanko JA , Balfour Sartor R , Fisher NC . T cell‐mediated oral tolerance is intact in germ‐free mice. Clin Exp Immunol. 2006;143:503‐512. 16487250 10.1111/j.1365-2249.2006.03019.x PMC1809622

[9] Sudo N , Sawamura S , Tanaka K , Aiba Y , Kubo C , Koga Y . The requirement of intestinal bacterial flora for the development of an IgE production system fully susceptible to oral tolerance induction. J Immunol. 1997;159:1739‐1745. 9257835

[10] Torii I , Shimizu S , Daimon T , et al. Exposure to high doses of lipopolysaccharide during ovalbumin sensitization prevents the development of allergic Th2 responses to a dietary antigen. J Toxicol Pathol. 2014;27:205‐215. 25378805 10.1293/tox.2014-0023 PMC4217231

[11] Zhang Y , Zhou X , Zhou B . DC‐derived TSLP promotes Th2 polarization in LPS‐primed allergic airway inflammation. Eur J Immunol. 2012;42:1735‐1743. 22585305 10.1002/eji.201142123 PMC3662367

[12] Zhou B , Comeau MR , Smedt TD , et al. Thymic stromal lymphopoietin as a key initiator of allergic airway inflammation in mice. Nat Immunol. 2005;6:1047‐1053. 16142237 10.1038/ni1247

[13] Comeau MR , Ziegler SF . The influence of TSLP on the allergic response. Mucosal Immunol. 2010;3:138‐147. 20016474 10.1038/mi.2009.134

[14] Fort MM , Cheung J , Yen D , et al. IL‐25 induces IL‐4, IL‐5, and IL‐13 and Th2‐associated pathologies in vivo. Immunity. 2001;15:985‐995. 11754819 10.1016/s1074-7613(01)00243-6

[15] Angkasekwinai P , Park H , Wang Y‐H , et al. Interleukin 25 promotes the initiation of proallergic type 2 responses. J Exp Med. 2007;204:1509‐1517. 17562814 10.1084/jem.20061675 PMC2118650

[16] Schmitz J , Owyang A , Oldham E , et al. IL‐33, an interleukin‐1‐like cytokine that signals via the IL‐1 receptor‐related protein ST2 and induces T helper type 2‐associated cytokines. Immunity. 2005;23:479‐490. 16286016 10.1016/j.immuni.2005.09.015

[17] Willart MA , Deswarte K , Pouliot P , et al. Interleukin‐1alpha controls allergic sensitization to inhaled house dust mite via the epithelial release of GM‐CSF and IL‐33. J Exp Med. 2012;209:1505‐1517. 22802353 10.1084/jem.20112691 PMC3409497

[18] Martinez‐Gonzalez I , Steer CA , Takei F . Lung ILC2s link innate and adaptive responses in allergic inflammation. Trends Immunol. 2015;36:189‐195. 25704560 10.1016/j.it.2015.01.005

[19] Klose CS , Artis D . Innate lymphoid cells as regulators of immunity, inflammation and tissue homeostasis. Nat Immunol. 2016;17:765‐774. 27328006 10.1038/ni.3489

[20] Lambrecht BN , Hammad H . The immunology of asthma. Nat Immunol. 2015;16:45‐56. 25521684 10.1038/ni.3049

[21] Lemaitre B , Nicolas E , Michaut L , Reichhart JM , Hoffmann JA . The dorsoventral regulatory gene cassette spatzle/Toll/cactus controls the potent antifungal response in Drosophila adults. Cell. 1996;86:973‐983. 8808632 10.1016/s0092-8674(00)80172-5

[22] Medzhitov R , Preston‐Hurlburt P , Janeway CA Jr . A human homologue of the Drosophila Toll protein signals activation of adaptive immunity. Nature. 1997;388:394‐397. 9237759 10.1038/41131

[23] Kawai T , Akira S . The role of pattern‐recognition receptors in innate immunity: update on Toll‐like receptors. Nat Immunol. 2010;11:373‐384. 20404851 10.1038/ni.1863

[24] Kawai T , Akira S . Toll‐like receptors and their crosstalk with other innate receptors in infection and immunity. Immunity. 2011;34:637‐650. 21616434 10.1016/j.immuni.2011.05.006

[25] Pandey S , Kawai T , Akira S . Microbial sensing by Toll‐like receptors and intracellular nucleic acid sensors. Cold Spring Harb Perspect Biol. 2014;7:a016246. 25301932 10.1101/cshperspect.a016246 PMC4292165

[26] Akira S , Uematsu S , Takeuchi O . Pathogen recognition and innate immunity. Cell. 2006;124:783‐801. 16497588 10.1016/j.cell.2006.02.015

[27] Lin SC , Lo YC , Wu H . Helical assembly in the MyD88‐IRAK4‐IRAK2 complex in TLR/IL‐1R signalling. Nature. 2010;465:885‐890. 20485341 10.1038/nature09121 PMC2888693

[28] Tesse R , Pandey RC , Kabesch M . Genetic variations in toll‐like receptor pathway genes influence asthma and atopy. Allergy. 2011;66:307‐316. 21039600 10.1111/j.1398-9995.2010.02489.x

[29] Lee E , Kwon JW , Kim HB , et al. Association between antibiotic exposure, bronchiolitis, and TLR4 (rs1927911) polymorphisms in childhood asthma. Allergy Asthma Immunol Res. 2015;7:167‐174. 25729624 10.4168/aair.2015.7.2.167 PMC4341338

[30] Xian M , Wawrzyniak P , Ruckert B , et al. Anionic surfactants and commercial detergents decrease tight junction barrier integrity in human keratinocytes. J Allergy Clin Immunol. 2016;138:890‐893. 27596709 10.1016/j.jaci.2016.07.003

[31] Guo S , Al‐Sadi R , Said HM , Ma TY . Lipopolysaccharide causes an increase in intestinal tight junction permeability in vitro and in vivo by inducing enterocyte membrane expression and localization of TLR‐4 and CD14. Am J Pathol. 2013;182:375‐387. 23201091 10.1016/j.ajpath.2012.10.014 PMC3562736

[32] Trompette A , Divanovic S , Visintin A , et al. Allergenicity resulting from functional mimicry of a Toll‐like receptor complex protein. Nature. 2009;457:585‐588. 19060881 10.1038/nature07548 PMC2843411

[33] Millien VO , Lu W , Shaw J , et al. Cleavage of fibrinogen by proteinases elicits allergic responses through Toll‐like receptor 4. Science. 2013;341:792‐796. 23950537 10.1126/science.1240342 PMC3898200

[34] Hammad H , Chieppa M , Perros F , Willart MA , Germain RN , Lambrecht BN . House dust mite allergen induces asthma via Toll‐like receptor 4 triggering of airway structural cells. Nat Med. 2009;15:410‐416. 19330007 10.1038/nm.1946 PMC2789255

[35] Noval Rivas M , Chatila TA . Regulatory T cells in allergic diseases. J Allergy Clin Immunol. 2016;138:639‐652. 27596705 10.1016/j.jaci.2016.06.003 PMC5023156

[36] Walker JA , McKenzie A . TH2 cell development and function. Nat Rev Immunol. 2018;18:121‐133. 29082915 10.1038/nri.2017.118

[37] Hennessy EJ , Parker AE , O'Neill LA . Targeting Toll‐like receptors: emerging therapeutics? Nat Rev Drug Discov. 2010;9:293‐307. 20380038 10.1038/nrd3203

[38] Zhu FG , Kandimalla ER , Yu D , Agrawal S . Oral administration of a synthetic agonist of Toll‐like receptor 9 potently modulates peanut‐induced allergy in mice. J Allergy Clin Immunol. 2007;120:631‐637. 17582479 10.1016/j.jaci.2007.05.015

[39] Aryan Z , Rezaei N . Toll‐like receptors as targets for allergen immunotherapy. Curr Opin Allergy Clin Immunol. 2015;15:568‐574. 26418475 10.1097/ACI.0000000000000212

[40] Tulic MK , Fiset P‐O , Christodoulopoulos P , et al. Amb a 1‐immunostimulatory oligodeoxynucleotide conjugate immunotherapy decreases the nasal inflammatory response. J Allergy Clin Immunol. 2004;113:235‐241. 14767435 10.1016/j.jaci.2003.11.001

[41] Creticos PS , Schroeder JT , Hamilton RG , et al. Immunotherapy with a ragweed‐toll‐like receptor 9 agonist vaccine for allergic rhinitis. N Engl J Med. 2006;355:1445‐1455. 17021320 10.1056/NEJMoa052916

[42] Senti G , Johansen P , Haug S , et al. Use of A‐type CpG oligodeoxynucleotides as an adjuvant in allergen‐specific immunotherapy in humans: a phase I/IIa clinical trial. Clin Exp Allergy. 2009;39:562‐570. 19226280 10.1111/j.1365-2222.2008.03191.x

[43] Aryan Z , Holgate ST , Radzioch D , Rezaei N . A new era of targeting the ancient gatekeepers of the immune system: toll‐like agonists in the treatment of allergic rhinitis and asthma. Int Arch Allergy Immunol. 2014;164:46‐63. 24853609 10.1159/000362553

[44] Dambuza IM , Brown GD . C‐type lectins in immunity: recent developments. Curr Opin Immunol. 2015;32:21‐27. 25553393 10.1016/j.coi.2014.12.002 PMC4589735

[45] Geijtenbeek TB , Gringhuis SI . C‐type lectin receptors in the control of T helper cell differentiation. Nat Rev Immunol. 2016;16:433‐448. 27291962 10.1038/nri.2016.55

[46] Hoving JC , Wilson GJ , Brown GD . Signalling C‐type lectin receptors, microbial recognition and immunity. Cell Microbiol. 2014;16:185‐194. 24330199 10.1111/cmi.12249 PMC4016756

[47] Zelensky AN , Gready JE . The C‐type lectin‐like domain superfamily. FEBS J. 2005;272:6179‐6217. 16336259 10.1111/j.1742-4658.2005.05031.x

[48] Saijo S , Iwakura Y . Dectin‐1 and Dectin‐2 in innate immunity against fungi. Int Immunol. 2011;23:467‐472. 21677049 10.1093/intimm/dxr046

[49] Plato A , Willment JA , Brown GD . C‐type lectin‐like receptors of the dectin‐1 cluster: ligands and signaling pathways. Int Rev Immunol. 2013;32:134‐156. 23570314 10.3109/08830185.2013.777065 PMC3634610

[50] Kerscher B , Willment JA , Brown GD . The Dectin‐2 family of C‐type lectin‐like receptors: an update. Int Immunol. 2013;25:271‐277. 23606632 10.1093/intimm/dxt006 PMC3631001

[51] Ishikawa E , Ishikawa T , Morita YS , et al. Direct recognition of the mycobacterial glycolipid, trehalose dimycolate, by C‐type lectin Mincle. J Exp Med. 2009;206:2879‐2888. 20008526 10.1084/jem.20091750 PMC2806462

[52] Behler‐Janbeck F , Takano T , Maus R , et al. C‐type lectin mincle recognizes glucosyl‐diacylglycerol of *Streptococcus pneumoniae* and plays a protective role in pneumococcal pneumonia. Plos Pathog. 2016;12:e1006038. 27923071 10.1371/journal.ppat.1006038 PMC5140071

[53] Imai T , Matsumura T , Mayer‐Lambertz S , et al. Lipoteichoic acid anchor triggers Mincle to drive protective immunity against invasive group A Streptococcus infection. Proc Natl Acad Sci U S A. 2018;115:E10662‐E10671. 30352847 10.1073/pnas.1809100115 PMC6233082

[54] Lu X , Nagata M , Yamasaki S . Mincle: 20 years of a versatile sensor of insults. Int Immunol. 2018;30:233‐239. 29726997 10.1093/intimm/dxy028

[55] Higashino‐Kameda M , Yabe‐Wada T , Matsuba S , et al. A critical role of Dectin‐1 in hypersensitivity pneumonitis. Inflamm Res. 2016;65:235‐244. 26644324 10.1007/s00011-015-0910-1

[56] Ito T , Hirose K , Norimoto A , et al. Dectin‐1 plays an important role in house dust mite‐induced allergic airway inflammation through the activation of CD11b+ dendritic cells. J Immunol. 2017;198:61‐70. 27852745 10.4049/jimmunol.1502393

[57] Overton NL , Simpson A , Bowyer P , Denning DW . Genetic susceptibility to severe asthma with fungal sensitization. Int J Immunogenet. 2017;44:93‐106. 28371335 10.1111/iji.12312

[58] Barrett NA , Maekawa A , Rahman OM , Austen KF , Kanaoka Y . Dectin‐2 recognition of house dust mite triggers cysteinyl leukotriene generation by dendritic cells. J Immunol. 2009;182:1119‐1128. 19124755 10.4049/jimmunol.182.2.1119 PMC3682801

[59] Barrett NA , Rahman OM , Fernandez JM , et al. Dectin‐2 mediates Th2 immunity through the generation of cysteinyl leukotrienes. J Exp Med. 2011;208:593‐604. 21357742 10.1084/jem.20100793 PMC3058587

[60] Parsons Mw , Li L , Wallace Am , et al. Dectin‐2 regulates the effector phase of house dust mite‐elicited pulmonary inflammation independently from its role in sensitization. J Immunol. 2014;192:1361‐1371. 24453247 10.4049/jimmunol.1301809 PMC4024442

[61] Kostarnoy AV , Gancheva PG , Lepenies B , et al. Receptor Mincle promotes skin allergies and is capable of recognizing cholesterol sulfate. Proc Natl Acad Sci U S A. 2017;114:E2758‐E2765. 28292894 10.1073/pnas.1611665114 PMC5380039

[62] Sirvent S , Soria I , Cirauqui C , et al. Novel vaccines targeting dendritic cells by coupling allergoids to nonoxidized mannan enhance allergen uptake and induce functional regulatory T cells through programmed death ligand 1. J Allergy Clin Immunol. 2016;138:558‐567. 27177779 10.1016/j.jaci.2016.02.029

[63] Soria I , López‐Relaño J , Viñuela M , et al. Oral myeloid cells uptake allergoids coupled to mannan driving Th1/Treg responses upon sublingual delivery in mice. Allergy. 2018;73:875‐884. 29319882 10.1111/all.13396 PMC5947296

[64] Benito‐Villalvilla C , Soria I , Subiza JL , Palomares O . Novel vaccines targeting dendritic cells by coupling allergoids to mannan. Allergo J Int. 2018;27:256‐262. 30546997 10.1007/s40629-018-0069-8 PMC6267119

[65] Edelmann KH , Richardson‐Burns S , Alexopoulou L , Tyler KL , Flavell RA , Oldstone MB . Does Toll‐like receptor 3 play a biological role in virus infections? Virology. 2004;322:231‐238. 15110521 10.1016/j.virol.2004.01.033

[66] Yoneyama M , Kikuchi M , Natsukawa T , et al. The RNA helicase RIG‐I has an essential function in double‐stranded RNA‐induced innate antiviral responses. Nat Immunol. 2004;5:730‐737. 15208624 10.1038/ni1087

[67] Andrejeva J , Childs KS , Young DF , et al. The V proteins of paramyxoviruses bind the IFN‐inducible RNA helicase, mda‐5, and inhibit its activation of the IFN‐beta promoter. Proc Natl Acad Sci U S A. 2004;101:17264‐17269. 15563593 10.1073/pnas.0407639101 PMC535396

[68] Yoneyama M , Fujita T . RNA recognition and signal transduction by RIG‐I‐like receptors. Immunol Rev. 2009;227:54‐65. 19120475 10.1111/j.1600-065X.2008.00727.x

[69] Kato H , Takahasi K , Fujita T . RIG‐I‐like receptors: cytoplasmic sensors for non‐self RNA. Immunol Rev. 2011;243:91‐98. 21884169 10.1111/j.1600-065X.2011.01052.x

[70] Kato H , Takeuchi O , Sato S , et al. Differential roles of MDA5 and RIG‐I helicases in the recognition of RNA viruses. Nature. 2006;441:101‐105. 16625202 10.1038/nature04734

[71] Pichlmair A , Schulz O , Tan Cp , et al. RIG‐I‐mediated antiviral responses to single‐stranded RNA bearing 5'‐phosphates. Science. 2006;314:997‐1001. 17038589 10.1126/science.1132998

[72] Hornung V , Ellegast J , Kim S , et al. 5'‐Triphosphate RNA is the ligand for RIG‐I. Science. 2006;314:994‐997. 17038590 10.1126/science.1132505

[73] Goubau D , Schlee M , Deddouche S , et al. Antiviral immunity via RIG‐I‐mediated recognition of RNA bearing 5'‐diphosphates. Nature. 2014;514:372‐375. 25119032 10.1038/nature13590 PMC4201573

[74] Kato H , Takeuchi O , Mikamo‐Satoh E , et al. Length‐dependent recognition of double‐stranded ribonucleic acids by retinoic acid‐inducible gene‐I and melanoma differentiation‐associated gene 5. J Exp Med. 2008;205:1601‐1610. 18591409 10.1084/jem.20080091 PMC2442638

[75] Seth RB , Sun L , Ea CK , Chen ZJ . Identification and characterization of MAVS, a mitochondrial antiviral signaling protein that activates NF‐kappaB and IRF 3. Cell. 2005;122:669‐682. 16125763 10.1016/j.cell.2005.08.012

[76] Meylan E , Curran J , Hofmann K , et al. Cardif is an adaptor protein in the RIG‐I antiviral pathway and is targeted by hepatitis C virus. Nature. 2005;437:1167‐1172. 16177806 10.1038/nature04193

[77] Kawai T , Takahashi K , Sato S , et al. IPS‐1, an adaptor triggering RIG‐I‐ and Mda5‐mediated type I interferon induction. Nat Immunol. 2005;6:981‐988. 16127453 10.1038/ni1243

[78] Xu LG , Wang YY , Han KJ , Li LY , Zhai Z , Shu HB . VISA is an adapter protein required for virus‐triggered IFN‐beta signaling. Mol Cell. 2005;19:727‐740. 16153868 10.1016/j.molcel.2005.08.014

[79] Hou F , Sun L , Zheng H , Skaug B , Jiang QX , Chen ZJ . MAVS forms functional prion‐like aggregates to activate and propagate antiviral innate immune response. Cell. 2011;146:448‐461. 21782231 10.1016/j.cell.2011.06.041 PMC3179916

[80] Holtzman MJ , Byers DE , Alexander‐Brett J , Wang X . The role of airway epithelial cells and innate immune cells in chronic respiratory disease. Nat Rev Immunol. 2014;14:686‐698. 25234144 10.1038/nri3739 PMC4782595

[81] Lamborn IT , Jing H , Zhang Yu , et al. Recurrent rhinovirus infections in a child with inherited MDA5 deficiency. J Exp Med. 2017;214:1949‐1972. 28606988 10.1084/jem.20161759 PMC5502429

[82] Asgari S , Schlapbach LJ , Anchisi S , et al. Severe viral respiratory infections in children with IFIH1 loss‐of‐function mutations. Proc Natl Acad Sci U S A. 2017;114:8342‐8347. 28716935 10.1073/pnas.1704259114 PMC5547624

[83] Diogo D , Tian C , Franklin CS , et al. Phenome‐wide association studies across large population cohorts support drug target validation. Nat Commun. 2018;9:4285. 30327483 10.1038/s41467-018-06540-3 PMC6191429

[84] Mahmutovic Persson I , Akbarshahi H , Menzel M , Brandelius A , Uller L . Increased expression of upstream TH2‐cytokines in a mouse model of viral‐induced asthma exacerbation. J Transl Med. 2016;14:52. 26879906 10.1186/s12967-016-0808-x PMC4754855

[85] Beale J , Jayaraman A , Jackson DJ , et al. Rhinovirus‐induced IL‐25 in asthma exacerbation drives type 2 immunity and allergic pulmonary inflammation. Sci Transl Med. 2014;6:256ra134. 10.1126/scitranslmed.3009124 PMC4246061 25273095

[86] Mahmutovic‐Persson I , Akbarshahi H , Bartlett Nw , et al. Inhaled dsRNA and rhinovirus evoke neutrophilic exacerbation and lung expression of thymic stromal lymphopoietin in allergic mice with established experimental asthma. Allergy. 2014;69:348‐358. 24283976 10.1111/all.12329 PMC4223976

[87] Luecke S , Holleufer A , Christensen MH , et al. cGAS is activated by DNA in a length‐dependent manner. Embo Rep. 2017;18:1707‐1715. 28801534 10.15252/embr.201744017 PMC5623850

[88] Ablasser A , Goldeck M , Cavlar T , et al. cGAS produces a 2'‐5'‐linked cyclic dinucleotide second messenger that activates STING. Nature. 2013;498:380‐384. 23722158 10.1038/nature12306 PMC4143541

[89] Diner E , Burdette D , Wilson S , et al. The innate immune DNA sensor cGAS produces a noncanonical cyclic dinucleotide that activates human STING. Cell Rep. 2013;3:1355‐1361. 23707065 10.1016/j.celrep.2013.05.009 PMC3706192

[90] Gao Pu , Ascano M , Wu Y , et al. Cyclic [G(2',5')pA(3',5')p] is the metazoan second messenger produced by DNA‐activated cyclic GMP‐AMP synthase. Cell. 2013;153:1094‐1107. 23647843 10.1016/j.cell.2013.04.046 PMC4382009

[91] Sun L , Wu J , Du F , Chen X , Chen ZJ . Cyclic GMP‐AMP synthase is a cytosolic DNA sensor that activates the type I interferon pathway. Science. 2013;339:786‐791. 23258413 10.1126/science.1232458 PMC3863629

[92] Wu J , Sun L , Chen X , et al. Cyclic GMP‐AMP is an endogenous second messenger in innate immune signaling by cytosolic DNA. Science. 2013;339:826‐830. 23258412 10.1126/science.1229963 PMC3855410

[93] Ishikawa H , Barber GN . STING is an endoplasmic reticulum adaptor that facilitates innate immune signalling. Nature. 2008;455:674‐678. 18724357 10.1038/nature07317 PMC2804933

[94] Zhong B , Yang Y , Li S , et al. The adaptor protein MITA links virus‐sensing receptors to IRF3 transcription factor activation. Immunity. 2008;29:538‐550. 18818105 10.1016/j.immuni.2008.09.003

[95] Sun W , Li Y , Chen L , et al. ERIS, an endoplasmic reticulum IFN stimulator, activates innate immune signaling through dimerization. Proc Natl Acad Sci U S A. 2009;106:8653‐8658. 19433799 10.1073/pnas.0900850106 PMC2689030

[96] Liu S , Cai X , Wu J , et al. Phosphorylation of innate immune adaptor proteins MAVS, STING, and TRIF induces IRF3 activation. Science. 2015;347:aaa2630. 25636800 10.1126/science.aaa2630

[97] Tanaka Y , Chen ZJ . STING specifies IRF3 phosphorylation by TBK1 in the cytosolic DNA signaling pathway. Sci Signal. 2012;5:ra20. 22394562 10.1126/scisignal.2002521 PMC3549669

[98] Gao D , Wu J , Wu Y‐t , et al. Cyclic GMP‐AMP synthase is an innate immune sensor of HIV and other retroviruses. Science. 2013;341:903‐906. 23929945 10.1126/science.1240933 PMC3860819

[99] Li XD , Wu J , Gao D , Wang H , Sun L , Chen ZJ . Pivotal roles of cGAS‐cGAMP signaling in antiviral defense and immune adjuvant effects. Science. 2013;341:1390‐1394. 23989956 10.1126/science.1244040 PMC3863637

[100] Mackenzie KJ , Carroll P , Martin C‐A , et al. cGAS surveillance of micronuclei links genome instability to innate immunity. Nature. 2017;548:461‐465. 28738408 10.1038/nature23449 PMC5870830

[101] Harding SM , Benci JL , Irianto J , Discher DE , Minn AJ , Greenberg RA . Mitotic progression following DNA damage enables pattern recognition within micronuclei. Nature. 2017;548:466‐470. 28759889 10.1038/nature23470 PMC5857357

[102] Chen G , Shaw MH , Kim YG , Nunez G . NOD‐like receptors: role in innate immunity and inflammatory disease. Annu Rev Pathol. 2009;4:365‐398. 18928408 10.1146/annurev.pathol.4.110807.092239

[103] Caruso R , Warner N , Inohara N , Nunez G . NOD1 and NOD2: signaling, host defense, and inflammatory disease. Immunity. 2014;41:898‐908. 25526305 10.1016/j.immuni.2014.12.010 PMC4272446

[104] Humphries F , Yang S , Wang B , Moynagh PN . RIP kinases: key decision makers in cell death and innate immunity. Cell Death Differ. 2015;22:225‐236. 25146926 10.1038/cdd.2014.126 PMC4291486

[105] Penack O , Smith OM , Cunningham‐Bussel A , et al. NOD2 regulates hematopoietic cell function during graft‐versus‐host disease. J Exp Med. 2009;206:2101‐2110. 19737867 10.1084/jem.20090623 PMC2757869

[106] Ogura Y , Lala S , Xin W , et al. Expression of NOD2 in Paneth cells: a possible link to Crohn's ileitis. Gut. 2003;52:1591‐1597. 14570728 10.1136/gut.52.11.1591 PMC1773866

[107] Pashenkov MV , Balyasova LS , Dagil YA , Pinegin BV . The role of the p38‐MNK‐eIF4E signaling axis in TNF production downstream of the NOD1 receptor. J Immunol. 2017;198:1638‐1648. 28087669 10.4049/jimmunol.1600467

[108] Rastogi R , Du W , Ju D , et al. Dysregulation of p38 and MKP‐1 in response to NOD1/TLR4 stimulation in sarcoid bronchoalveolar cells. Am J Respir Crit Care Med. 2011;183:500‐510. 20851927 10.1164/rccm.201005-0792OC PMC5450927

[109] Fritz JH , Le Bourhis L , Sellge G , et al. Nod1‐mediated innate immune recognition of peptidoglycan contributes to the onset of adaptive immunity. Immunity. 2007;26:445‐459. 17433730 10.1016/j.immuni.2007.03.009

[110] Duan W , Mehta AK , Magalhaes JG , et al. Innate signals from Nod2 block respiratory tolerance and program T(H)2‐driven allergic inflammation. J Allergy Clin Immunol. 2010;126:1284‐1293. 21051079 10.1016/j.jaci.2010.09.021 PMC3058679

[111] Ramasamy A , Curjuric I , Coin LJ , et al. A genome‐wide meta‐analysis of genetic variants associated with allergic rhinitis and grass sensitization and their interaction with birth order. J Allergy Clin Immunol. 2011;128:996‐1005. 22036096 10.1016/j.jaci.2011.08.030

[112] Hysi P , Kabesch M , Moffatt MF , et al. NOD1 variation, immunoglobulin E and asthma. Hum Mol Genet. 2005;14:935‐941. 15718249 10.1093/hmg/ddi087

[113] Macaluso F , Nothnagel M , Parwez Q , et al. Polymorphisms in NACHT‐LRR (NLR) genes in atopic dermatitis. Exp Dermatol. 2007;16:692‐698. 17620097 10.1111/j.1600-0625.2007.00589.x

[114] Bursztejn Ac , Romano A , Guéant‐Rodriguez Rm , et al. Allergy to betalactams and nucleotide‐binding oligomerization domain (NOD) gene polymorphisms. Allergy. 2013;68:1076‐1080. 23888881 10.1111/all.12196

[115] Kabesch M , Peters W , Carr D , Leupold W , Weiland SK , von Mutius E . Association between polymorphisms in caspase recruitment domain containing protein 15 and allergy in two German populations. J Allergy Clin Immunol. 2003;111:813‐817. 12704363 10.1067/mai.2003.1336

[116] Keestra‐Gounder AM , Byndloss MX , Seyffert N , et al. NOD1 and NOD2 signalling links ER stress with inflammation. Nature. 2016;532:394‐397. 27007849 10.1038/nature17631 PMC4869892

[117] Broz P , Dixit VM . Inflammasomes: mechanism of assembly, regulation and signalling. Nat Rev Immunol. 2016;16:407‐420. 27291964 10.1038/nri.2016.58

[118] Dorfleutner A , Chu L , Stehlik C . Inhibiting the inflammasome: one domain at a time. Immunol Rev. 2015;265:205‐216. 25879295 10.1111/imr.12290 PMC4400809

[119] Indramohan M , Stehlik C , Dorfleutner A . COPs and POPs patrol inflammasome activation. J Mol Biol. 2018;430:153‐173. 29024695 10.1016/j.jmb.2017.10.004 PMC5766406

[120] Man SM , Kanneganti TD . Regulation of inflammasome activation. Immunol Rev. 2015;265:6‐21. 25879280 10.1111/imr.12296 PMC4400844

[121] Franchi L , Munoz‐Planillo R , Nunez G . Sensing and reacting to microbes through the inflammasomes. Nat Immunol. 2012;13:325‐332. 22430785 10.1038/ni.2231 PMC3449002

[122] Cookson BT , Brennan MA . Pro‐inflammatory programmed cell death. Trends Microbiol. 2001;9:113‐114. 11303500 10.1016/s0966-842x(00)01936-3

[123] Nadiri A , Wolinski MK , Saleh M . The inflammatory caspases: key players in the host response to pathogenic invasion and sepsis. J Immunol. 2006;177:4239‐4245. 16982854 10.4049/jimmunol.177.7.4239

[124] He Y , Hara H , Nunez G . Mechanism and regulation of NLRP3 inflammasome activation. Trends Biochem Sci. 2016;41:1012‐1021. 27669650 10.1016/j.tibs.2016.09.002 PMC5123939

[125] Chen GY , Nunez G . Sterile inflammation: sensing and reacting to damage. Nat Rev Immunol. 2010;10:826‐837. 21088683 10.1038/nri2873 PMC3114424

[126] Abais JM , Xia M , Zhang Y , Boini KM , Li PL . Redox regulation of NLRP3 inflammasomes: ROS as trigger or effector? Antioxid Redox Signal. 2015;22:1111‐1129. 25330206 10.1089/ars.2014.5994 PMC4403231

[127] Netea MG , van de Veerdonk FL , van der Meer JW , Dinarello CA , Joosten LA . Inflammasome‐independent regulation of IL‐1‐family cytokines. Annu Rev Immunol. 2015;33:49‐77. 25493334 10.1146/annurev-immunol-032414-112306

[128] Carta S , Penco F , Lavieri R , et al. Cell stress increases ATP release in NLRP3 inflammasome‐mediated autoinflammatory diseases, resulting in cytokine imbalance. Proc Natl Acad Sci U S A. 2015;112:2835‐2840. 25730877 10.1073/pnas.1424741112 PMC4352822

[129] Chung Y , Chang SH , Martinez GJ , et al. Critical regulation of early Th17 cell differentiation by interleukin‐1 signaling. Immunity. 2009;30:576‐587. 19362022 10.1016/j.immuni.2009.02.007 PMC2705871

[130] Banuelos J , Cao Y , Shin SC , Lu NZ . Immunopathology alters Th17 cell glucocorticoid sensitivity. Allergy. 2017;72:331‐341. 27646878 10.1111/all.13051 PMC5315659

[131] Xiao Y , Xu W , Su W . NLRP3 inflammasome: a likely target for the treatment of allergic diseases. Clin Exp Allergy. 2018;48:1080‐1091. 29900602 10.1111/cea.13190

[132] Choy DF , Hart KM , Borthwick LA , et al. TH2 and TH17 inflammatory pathways are reciprocally regulated in asthma. Sci Transl Med. 2015;7:301ra129. 10.1126/scitranslmed.aab3142 26290411

[133] Unterholzner L , Keating SE , Baran M , et al. IFI16 is an innate immune sensor for intracellular DNA. Nat Immunol. 2010;11:997‐1004. 20890285 10.1038/ni.1932 PMC3142795

[134] Jin T , Perry A , Jiang J , et al. Structures of the HIN domain:DNA complexes reveal ligand binding and activation mechanisms of the AIM2 inflammasome and IFI16 receptor. Immunity. 2012;36:561‐571. 22483801 10.1016/j.immuni.2012.02.014 PMC3334467

[135] Jin T , Perry A , Smith P , Jiang J , Xiao TS . Structure of the absent in melanoma 2 (AIM2) pyrin domain provides insights into the mechanisms of AIM2 autoinhibition and inflammasome assembly. J Biol Chem. 2013;288:13225‐13235. 23530044 10.1074/jbc.M113.468033 PMC3650362

[136] Hornung V , Ablasser A , Charrel‐Dennis M , et al. AIM2 recognizes cytosolic dsDNA and forms a caspase‐1‐activating inflammasome with ASC. Nature. 2009;458:514‐518. 19158675 10.1038/nature07725 PMC2726264

[137] Fernandes‐Alnemri T , Yu JW , Datta P , Wu J , Alnemri ES . AIM2 activates the inflammasome and cell death in response to cytoplasmic DNA. Nature. 2009;458:509‐513. 19158676 10.1038/nature07710 PMC2862225

[138] Bürckstümmer T , Baumann C , Blüml S , et al. An orthogonal proteomic‐genomic screen identifies AIM2 as a cytoplasmic DNA sensor for the inflammasome. Nat Immunol. 2009;10:266‐272. 19158679 10.1038/ni.1702

[139] Roberts Tl , Idris A , Dunn Ja , et al. HIN‐200 proteins regulate caspase activation in response to foreign cytoplasmic DNA. Science. 2009;323:1057‐1060. 19131592 10.1126/science.1169841

[140] Man SM , Karki R , Kanneganti TD . AIM2 inflammasome in infection, cancer, and autoimmunity: role in DNA sensing, inflammation, and innate immunity. Eur J Immunol. 2016;46:269‐280. 26626159 10.1002/eji.201545839 PMC4758349

[141] Su S , Zhao J , Xing Y , et al. Immune checkpoint inhibition overcomes ADCP‐induced immunosuppression by macrophages. Cell. 2018;175:442‐457. 30290143 10.1016/j.cell.2018.09.007

[142] Wang Y , Ning X , Gao P , et al. Inflammasome activation triggers caspase‐1‐mediated cleavage of cGAS to regulate responses to DNA virus infection. Immunity. 2017;46:393‐404. 28314590 10.1016/j.immuni.2017.02.011

[143] Gaidt MM , Ebert TS , Chauhan D , et al. The DNA inflammasome in human myeloid cells is initiated by a STING‐cell death program upstream of NLRP3. Cell. 2017;171:1110‐1124. 29033128 10.1016/j.cell.2017.09.039 PMC5901709

[144] Swanson KV , Junkins RD , Kurkjian CJ , et al. A noncanonical function of cGAMP in inflammasome priming and activation. J Exp Med. 2017;214:3611‐3626. 29030458 10.1084/jem.20171749 PMC5716045

[145] Duncan JA , Canna SW . The NLRC4 inflammasome. Immunol Rev. 2018;281:115‐123. 29247997 10.1111/imr.12607 PMC5897049

[146] Reyes Ruiz VM , Ramirez J , Naseer N , et al. Broad detection of bacterial type III secretion system and flagellin proteins by the human NAIP/NLRC4 inflammasome. Proc Natl Acad Sci U S A. 2017;114:13242‐13247. 29180436 10.1073/pnas.1710433114 PMC5740664

[147] Kofoed EM , Vance RE . Innate immune recognition of bacterial ligands by NAIPs determines inflammasome specificity. Nature. 2011;477:592‐595. 21874021 10.1038/nature10394 PMC3184209

[148] Zhao Y , Yang J , Shi J , et al. The NLRC4 inflammasome receptors for bacterial flagellin and type III secretion apparatus. Nature. 2011;477:596‐600. 21918512 10.1038/nature10510

[149] Zhao Y , Shi J , Shi X , Wang Y , Wang F , Shao F . Genetic functions of the NAIP family of inflammasome receptors for bacterial ligands in mice. J Exp Med. 2016;213:647‐656. 27114610 10.1084/jem.20160006 PMC4854738

[150] Rauch I , Tenthorey JL , Nichols RD , et al. NAIP proteins are required for cytosolic detection of specific bacterial ligands in vivo. J Exp Med. 2016;213:657‐665. 27045008 10.1084/jem.20151809 PMC4854734

[151] Karki R , Lee E , Place D , et al. IRF8 regulates transcription of naips for NLRC4 inflammasome activation. Cell. 2018;173:920‐933. 29576451 10.1016/j.cell.2018.02.055 PMC5935577

[152] Hiruma J , Harada K , Motoyama A , et al. Key component of inflammasome, NLRC4, was identified in the lesional epidermis of psoriatic patients. J Dermatol. 2018;45:971‐977. 29797527 10.1111/1346-8138.14478

[153] Uh ST , Park JS , Koo SM , et al. Association of genetic variants of NLRP4 with exacerbation of asthma: the effect of smoking. DNA Cell Biol. 2019;38:76‐84. 30526007 10.1089/dna.2018.4433

[154] Rathinam V , Vanaja S , Waggoner L , et al. TRIF licenses caspase‐11‐dependent NLRP3 inflammasome activation by gram‐negative bacteria. Cell. 2012;150:606‐619. 22819539 10.1016/j.cell.2012.07.007 PMC3660860

[155] Kayagaki N , Warming S , Lamkanfi M , et al. Non‐canonical inflammasome activation targets caspase‐11. Nature. 2011;479:117‐121. 22002608 10.1038/nature10558

[156] Akhter A , Caution K , Abu Khweek A , et al. Caspase‐11 promotes the fusion of phagosomes harboring pathogenic bacteria with lysosomes by modulating actin polymerization. Immunity. 2012;37:35‐47. 22658523 10.1016/j.immuni.2012.05.001 PMC3408798

[157] Case Cl , Kohler Lj , Lima Jb , et al. Caspase‐11 stimulates rapid flagellin‐independent pyroptosis in response to *Legionella pneumophila* . Proc Natl Acad Sci U S A. 2013;110:1851‐1856. 23307811 10.1073/pnas.1211521110 PMC3562791

[158] Aachoui Y , Leaf Ia , Hagar Ja , et al. Caspase‐11 protects against bacteria that escape the vacuole. Science. 2013;339:975‐978. 23348507 10.1126/science.1230751 PMC3697099

[159] Hagar JA , Powell DA , Aachoui Y , Ernst RK , Miao EA . Cytoplasmic LPS activates caspase‐11: implications in TLR4‐independent endotoxic shock. Science. 2013;341:1250‐1253. 24031018 10.1126/science.1240988 PMC3931427

[160] Kayagaki N , Wong Mt , Stowe Ib , et al. Noncanonical inflammasome activation by intracellular LPS independent of TLR4. Science. 2013;341:1246‐1249. 23887873 10.1126/science.1240248

[161] Shi J , Zhao Y , Wang Y , et al. Inflammatory caspases are innate immune receptors for intracellular LPS. Nature. 2014;514:187‐192. 25119034 10.1038/nature13683

[162] Stowe I , Lee B , Kayagaki N . Caspase‐11: arming the guards against bacterial infection. Immunol Rev. 2015;265:75‐84. 25879285 10.1111/imr.12292

[163] Hara H , Seregin SS , Yang D , et al. The NLRP6 inflammasome recognizes lipoteichoic acid and regulates gram‐positive pathogen infection. Cell. 2018;175:1651‐1664. 30392956 10.1016/j.cell.2018.09.047 PMC6294477

[164] Rieber N , Singh A , Öz H , et al. Pathogenic fungi regulate immunity by inducing neutrophilic myeloid‐derived suppressor cells. Cell Host Microbe. 2015;17:507‐514. 25771792 10.1016/j.chom.2015.02.007 PMC4400268

[165] Gringhuis SI , Kaptein TM , Wevers BA , et al. Dectin‐1 is an extracellular pathogen sensor for the induction and processing of IL‐1beta via a noncanonical caspase‐8 inflammasome. Nat Immunol. 2012;13:246‐254. 22267217 10.1038/ni.2222

[166] Ganesan S , Rathinam V , Bossaller L , et al. Caspase‐8 modulates dectin‐1 and complement receptor 3‐driven IL‐1beta production in response to beta‐glucans and the fungal pathogen, *Candida albicans* . J Immunol. 2014;193:2519‐2530. 25063877 10.4049/jimmunol.1400276 PMC4134963

[167] Gurung P , Anand Pk , Malireddi R , et al. FADD and caspase‐8 mediate priming and activation of the canonical and noncanonical Nlrp3 inflammasomes. J Immunol. 2014;192:1835‐1846. 24453255 10.4049/jimmunol.1302839 PMC3933570

[168] Qi X , Gurung P , Malireddi R , et al. Critical role of caspase‐8‐mediated IL‐1 signaling in promoting Th2 responses during asthma pathogenesis. Mucosal Immunol. 2017;10:128‐138. 27007676 10.1038/mi.2016.25 PMC5035164

[169] Zhong FL , Mamai O , Sborgi L , et al. Germline NLRP1 mutations cause skin inflammatory and cancer susceptibility syndromes via inflammasome activation. Cell. 2016;167:187‐202. 27662089 10.1016/j.cell.2016.09.001

[170] Cunha LD , Ribeiro JM , Fernandes TD , et al. Inhibition of inflammasome activation by Coxiella burnetii type IV secretion system effector IcaA. Nat Commun. 2015;6:10205. 26687278 10.1038/ncomms10205 PMC4703858

[171] Malireddi R , Gurung P , Mavuluri J , et al. TAK1 restricts spontaneous NLRP3 activation and cell death to control myeloid proliferation. J Exp Med. 2018;215:1023‐1034. 29500178 10.1084/jem.20171922 PMC5881469

[172] Orning P , Weng D , Starheim K , et al. Pathogen blockade of TAK1 triggers caspase‐8‐dependent cleavage of gasdermin D and cell death. Science. 2018;362:1064‐1069. 30361383 10.1126/science.aau2818 PMC6522129

[173] Xu D , Jin T , Zhu H , et al. TBK1 suppresses RIPK1‐driven apoptosis and inflammation during development and in aging. Cell. 2018;174:1477‐1491. 30146158 10.1016/j.cell.2018.07.041 PMC6128749

[174] Lamkanfi M , Dixit VM . Manipulation of host cell death pathways during microbial infections. Cell Host Microbe. 2010;8:44‐54. 20638641 10.1016/j.chom.2010.06.007

[175] Brinkmann V , Reichard U , Goosmann C , et al. Neutrophil extracellular traps kill bacteria. Science. 2004;303:1532‐1535. 15001782 10.1126/science.1092385

[176] Jorch SK , Kubes P . An emerging role for neutrophil extracellular traps in noninfectious disease. Nat Med. 2017;23:279‐287. 28267716 10.1038/nm.4294

[177] Toussaint M , Jackson DJ , Swieboda D , et al. Host DNA released by NETosis promotes rhinovirus‐induced type‐2 allergic asthma exacerbation. Nat Med. 2017;23:681‐691. 28459437 10.1038/nm.4332 PMC5821220

[178] Toussaint M , Jackson DJ , Swieboda D , et al. Corrigendum: host DNA released by NETosis promotes rhinovirus‐induced type‐2 allergic asthma exacerbation. Nat Med. 2017;23:1384. 10.1038/nm1117-1384a 29117172

[179] Feng S , Fox D , Man SM . Mechanisms of gasdermin family members in inflammasome signaling and cell death. J Mol Biol. 2018;430:3068‐3080. 29990470 10.1016/j.jmb.2018.07.002

[180] Shi J , Zhao Y , Wang K , et al. Cleavage of GSDMD by inflammatory caspases determines pyroptotic cell death. Nature. 2015;526:660‐665. 26375003 10.1038/nature15514

[181] Liu X , Zhang Z , Ruan J , et al. Inflammasome‐activated gasdermin D causes pyroptosis by forming membrane pores. Nature. 2016;535:153‐158. 27383986 10.1038/nature18629 PMC5539988

[182] He WT , Wan H , Hu L , et al. Gasdermin D is an executor of pyroptosis and required for interleukin‐1beta secretion. Cell Res. 2015;25:1285‐1298. 26611636 10.1038/cr.2015.139 PMC4670995

[183] Ding J , Wang K , Liu W , et al. Pore‐forming activity and structural autoinhibition of the gasdermin family. Nature. 2016;535:111‐116. 27281216 10.1038/nature18590

[184] Aglietti RA , Estevez A , Gupta A , et al. GsdmD p30 elicited by caspase‐11 during pyroptosis forms pores in membranes. Proc Natl Acad Sci U S A. 2016;113:7858‐7863. 27339137 10.1073/pnas.1607769113 PMC4948338

[185] Sborgi L , Rühl S , Mulvihill E , et al. GSDMD membrane pore formation constitutes the mechanism of pyroptotic cell death. EMBO J. 2016;35:1766‐1778. 27418190 10.15252/embj.201694696 PMC5010048

[186] Chen X , He W‐T , Hu L , et al. Pyroptosis is driven by non‐selective gasdermin‐D pore and its morphology is different from MLKL channel‐mediated necroptosis. Cell Res. 2016;26:1007‐1020. 27573174 10.1038/cr.2016.100 PMC5034106

[187] Sollberger G , Choidas A , Burn GL , et al. Gasdermin D plays a vital role in the generation of neutrophil extracellular traps. Sci Immunol. 2018;3:eaar6689. 30143555 10.1126/sciimmunol.aar6689

[188] Rathkey JK , Zhao J , Liu Z , et al. Chemical disruption of the pyroptotic pore‐forming protein gasdermin D inhibits inflammatory cell death and sepsis. Sci Immunol. 2018;3:eaat2738. 30143556 10.1126/sciimmunol.aat2738 PMC6462819

[189] Chen KW , Monteleone M , Boucher D , et al. Noncanonical inflammasome signaling elicits gasdermin D‐dependent neutrophil extracellular traps. Sci Immunol. 2018;3:eaar6676. 30143554 10.1126/sciimmunol.aar6676

[190] Moffatt MF , Kabesch M , Liang L , et al. Genetic variants regulating ORMDL3 expression contribute to the risk of childhood asthma. Nature. 2007;448:470‐473. 17611496 10.1038/nature06014

[191] Moffatt MF , Gut IG , Demenais F , et al. A large‐scale, consortium‐based genomewide association study of asthma. N Engl J Med. 2010;363:1211‐1221. 20860503 10.1056/NEJMoa0906312 PMC4260321

[192] Das S , Miller M , Beppu AK , et al. GSDMB induces an asthma phenotype characterized by increased airway responsiveness and remodeling without lung inflammation. Proc Natl Acad Sci U S A. 2016;113:13132‐13137. 27799535 10.1073/pnas.1610433113 PMC5135378

[193] Gaudet Rg , Sintsova A , Buckwalter Cm , et al. Cytosolic detection of the bacterial metabolite HBP activates TIFA‐dependent innate immunity. Science. 2015;348:1251‐1255. 26068852 10.1126/science.aaa4921

[194] Milivojevic M , Dangeard AS , Kasper CA , et al. ALPK1 controls TIFA/TRAF6‐dependent innate immunity against heptose‐1,7‐bisphosphate of gram‐negative bacteria. Plos Pathog. 2017;13:e1006224. 28222186 10.1371/journal.ppat.1006224 PMC5336308

[195] Zimmermann S , Pfannkuch L , Al‐Zeer MA , et al. ALPK1‐ and TIFA‐dependent innate immune response triggered by the *Helicobacter pylori* type IV secretion system. Cell Rep. 2017;20:2384‐2395. 28877472 10.1016/j.celrep.2017.08.039

[196] Kanamori M , Suzuki H , Saito R , Muramatsu M , Hayashizaki Y . T2BP, a novel TRAF2 binding protein, can activate NF‐kappaB and AP‐1 without TNF stimulation. Biochem Biophys Res Commun. 2002;290:1108‐1113. 11798190 10.1006/bbrc.2001.6315

[197] Zhou P , She Y , Dong Na , et al. Alpha‐kinase 1 is a cytosolic innate immune receptor for bacterial ADP‐heptose. Nature. 2018;561:122‐126. 30111836 10.1038/s41586-018-0433-3

[198] García‐Weber D , Dangeard AS , Cornil J , et al. ADP‐heptose is a newly identified pathogen‐associated molecular pattern of *Shigella flexneri* . EMBO Rep. 2018;19:e46943. 30455202 10.15252/embr.201846943 PMC6280651

